# A review of *Biston* Leach, 1815 (Lepidoptera, Geometridae, Ennominae) from China, with description of one new species

**DOI:** 10.3897/zookeys.139.1308

**Published:** 2011-10-25

**Authors:** Nan Jiang, Dayong Xue, Hongxiang Han

**Affiliations:** 1 Key Laboratory of Zoological Systematics and Evolution, Institute of Zoology, Chinese Academy of Sciences, Beijing 100101, China; 2 Graduate University of Chinese Academy of Sciences, Beijing 100049, China

**Keywords:** *Biston*, taxonomy, new species, Geometridae, Lepidoptera

## Abstract

The genus *Biston* Leach, 1815 is reviewed for China. Seventeen species are recognized, of which *Biston mediolata* **sp. n.** is described. *Biston pustulata* (Warren, 1896) and *Biston panterinaria* *exanthemata* (Moore, 1888) are newly recorded for China. The following new synonyms are established: *Biston suppressaria suppressaria* (Guenée, 1858) (= *Biston suppressaria benescripta* (Prout, 1915), **syn. n.** = *Biston luculentus* Inoue, 1992 **syn. n.**); *Biston falcata* (Warren, 1893) (= *Amphidasis* *erilda* Oberthür, 1910, **syn. n.** = *Amphidasis clorinda* Oberthür, 1910, **syn. n.** = *Biston emarginaria* Leech, 1897, **syn. n.**); *Biston panterinaria panterinaria* (Bremer & Grey, 1853) (= *Biston panterinaria abraxata* (Leech, 1889), **syn. n.** = *Biston panterinaria lienpingensis* (Wehrli, 1939), **syn. n.** = B*. panterinaria szechuanensis* (Wehrli, 1939), **syn. n.**). *Biston falcata satura* (Wehrli,1941), **comb. n.** is proposed. A key to Chinese *Biston* and diagnoses for Chinese species are provided. Illustrations of external features and genitalia are presented.

## Introduction

Leach (1815) established the genus *Biston* Leach, 1815 with three species,   *Geometra* *prodromaria* Denis & Schiffermüller, 1775, *Phalaena (Geometra) betularia* Linnaeus, 1758 and *Phalaena hirtaria* Clerck, 1759. *Geometra prodromaria* was designated as the type species of *Biston* by Westwood (1840) and was later found to be a junior synonym of *Phalaena strataria* (Hufnagel, 1767) by Prout (1915). *Phalaena hirtaria* was designated as the type species of *Lycia* Hübner, 1825 by Hulst (1896). *Phalaena betularia* is still treated as a member of *Biston*. Hampson (1895), as the first author, presented a wider concept of *Biston* and included *Eubyjodonta* Warren, 1893, *Amraica* Moore, 1888, *Buzura* Walker, 1863 and *Cusiala* Moore, 1887 in the genus *Biston*. Prout (1915) also included the species of *Eubyjodonta* in *Biston*, but did not explicitly state *Eubyjodonta* as a synonym. Subsequently, this genus was considered as a subgenus of *Biston* by Wehrli (1941). In addition, Prout (1915) treated *Buzura* as a separate genus, and regarded *Amraica* and *Blepharoctenia* Warren, 1894 as different sections of *Buzura* according to the structure of male antennae, and moved *Cusiala* to his very broadly defined “genus” *Boarmia*. (Inoue (1982a, 1985) established the broader concept of *Biston* by examining external and genital characters of the Palaearctic and the East Asiatic species. He treated *Buzura* as a junior synonym of *Biston* and raised *Amraica* to generic level. Sato (1996) also considered *Culcula* Moore, 1888 as a junior synonym of *Biston*. Parsons et al. (1999) summarized the previous works, and besides the generic names mentioned above, they also included *Epamraica* Matsumura, 1910 and *Eubyja* Hübner, 1825 as junior synonyms of *Biston*. (Rindge (1975, 1985) summerized New World Bistonini and gave a valuable diagnosis for the genus *Biston*.

Holloway (1994) proposed a very broad concept of the tribe Boarmiini which also subsumed the previously separate tribe Bistonini, and provided the diagnostic characters for the genus *Biston*.

*Biston* indeed has some typical features in common with the Boarmiini: the postmedial lines of both wings often protrudes outwards between M_1_ and M_3_; in the male genitalia, the socii are usually absent; the valva has a strong cucullus. However, *Biston* also has some features atypical for Boarmiini: a fovea is absent in the male forewing; in the male genitalia, the valva is simple, without any ornamentation (Holloway 1994; Pitkin 2002; Viidalepp et al. 2007; Young 2008).

The species of *Biston* are widely distributed in Holarctic, Oriental and Ethiopian regions. Reviews of the genus are available for some geographical regions, e.g. for Africa (Karisch 2005), for North America (Rindge 1975, 1985) and partly for Asia (Holloway 1994; Viidalepp 1979, 2003; Yazaki 1992; Sato 1996; Inoue 1982a, 1982b, 1985, 1992, 2000; Ghosh 2003). Parsons et al. (1999) listed 50 species and 38 subspecies. Sato (2003) placed *Biston praeparva* (Prout, 1937) and *Biston semifusca* (Swinhoe, 1902) into the genus *Amraica*. Until now, 54 species and 40 subspecies in the genus *Biston* have been recognized, with 17 species and 14 subspecies recorded in China. Major contributions concerned with the Chinese fauna of the genus have been published by Bremer and Grey (1853), (Oberthür (1884, 1886, 1910), (Leech (1889, 1897), Hampsom (1895), Warren (1899), Bastelberger (1909), Prout (1915), Wehrli (1938–1954), (Inoue (1964, 1982b), (Zhu (1981, 1982), Heppner and Inoue (1992), Zhu and Xue (1992, 1998), Xue (1992a, 1992b, 1993, 1997, 2001), Sato (1996), Wang (1998), Xue et al. (2002), Han and Xue (2002, 2004), Ades and Kendrick (2004), Hua (2005), Xue and Han (2005). However, it has become apparent that, with the study of the material obtained during recent expeditions and the re-examination of the IZCAS collection, a new species needs to be described, the taxonomy needs to be revised, and the Chinese *Biston* fauna needs to be summarized.

Here, we divide the Chinese *Biston* into three species groups based on morphological characters. Group I includes the “typical” species of *Biston*. Group II includes *Biston brevipennata* Inoue, 1982 and the species which were treated in the subgenus *Eubyjodonta* of *Biston* by Wehrli (1941). Group III includes *Biston perclara* (Warren, 1899), *Biston thibetaria* (Oberthür, 1886) and *Biston panterinaria* (Bremer & Grey, 1853) which was considered slightly different from the typical species of *Biston* by Sato (1996).

The purpose of this paper is, to review all known Chinese *Biston* species, to determine their diagnostic characters, to develope a key for their determination and to provide illustrations of external features and genitalia; furthermore, one new species, *Biston mediolata* sp. n., will be described, *Biston pustulata* (Warren, 1896) will be recorded as new for the fauna of China and several new synonyms and a new combination will be proposed. This results, to our present knowledge, in 17 species and nine subspecies of *Biston* for the fauna of China and 52 species with 33 subspecies worldwide.

## Material and methods

Specimens of *Biston* were obtained from Institute of Zoology, Chinese Academy of Sciences, Beijing, China (IZCAS) and Zoologisches Forschungsmuseum Alexander Koenig, Bonn, Germany (ZFMK). The other museums cited here, where types are deposited, are the Natural History Museum, London, United Kingdom (BMNH), the Linnean Society of London, United Kingdom (LSL), the Zoologische Staatssammlung Muenchen, Munich, Germany (ZSM) and the Zoological Institute, Russian Academy of Sciences, Saint-Petersburg, Russia (ZISP). Terminology for wing venation followed the Comstock-Needham System (Comstock 1918) as adopted for Geometridae by Scoble (1992) and Hausmann (2001), and that of the genitalia was based on Pierce (1914), Klots (1970) and Nichols (1989). Photographs of adult moths and their genitalia were taken with digital cameras. Composite images were generated using Auto-Montage software version 5.03.0061 (Synoptics Ltd). The plates were compiled using Adobe Photoshop software.

## Taxonomic account

### Biston

Leach, 1815

http://species-id.net/wiki/Biston

Biston Leach, 1815, *Brewster’s Edinburgh Encyclopaedia*, 9: 134. Type species: *Geometra prodromaria*Denis & Schiffermüller, 1775 (= *Phalaena strataria* (Hufnagel, 1767)), by subsequent designation by Westwood, 1840.
Dasyphara Billberg, 1820, *Enumeratio Insect. Mus. G. J. Billberg*: 89. Type species: *Geometra prodromaria*Denis & Schiffermüller, 1775. [Junior objective synonym of *Biston* Leach.]
Pachys Hübner, 1822, *Syst.-alphab. Verz.*: 38*–*44, 46, 47, 49, 50, 52. Type species: *Geometra prodromaria*Denis & Schiffermüller, 1775. [Junior objective synonym of Biston Leach.]
Eubyja Hübner, 1825, *Verz. bekannter Schmett.*: 318. Type species: *Phalaena betularia* Linnaeus, 1758, by subsequent designation by Grote, 1902.
Amphidasis Treitschke, 1825, *in* Ochsenheimer, *Schmett. Eur.*, 5 (2): 434. Type species: *Geometra prodromaria*Denis & Schiffermüller, 1775. [Junior objective synonym of *Biston* Leach.]
Amphidasys Sodoffsky, 1837, *Bull. Soc. imp. Nat. Moscou*, 10: 90. [Emendation of *Amphidasis* Treitschke.]
Amphidasea Unger, 1856, *Arch. Ver. Freunde Naturg.-Mecklenb.*, 10: 61. [Emendation of *Amphidasis* Treitschke.]
Buzura Walker, 1863, *List Specimens lepid. Insects Colln Br. Mus.*, 26: 1531. Type species: *Buzura multipunctaria* Walker, 1863, by monotypy.
Culcula Moore, 1888, *in* Hewitson & Moore, *Descr. new Indian lepid. Insects Colln late Mr W.S. Atkinson*, (3): 266. Type species: *Culcula exanthemata* Moore, 1888, by monotypy.
Eubyjodonta Warren, 1893, *Proc. zool. Soc. Lond.*, 1893: 416. Type species: *Eubyjodonta falcata* Warren, 1893, by original designation.
Blepharoctenia Warren, 1894, *Novit. zool.*, 1: 428. Type species: *Amphidasys bengaliaria*Guenée, 1858, by original designation.
Epamraica Matsumura, 1910, *Thousand Insects Japan*, (Suppl.) 2: 130. Type species: *Epamraica bilineata* Matsumura, 1910, by monotypy.

#### Description.

**Figures 1–3.**
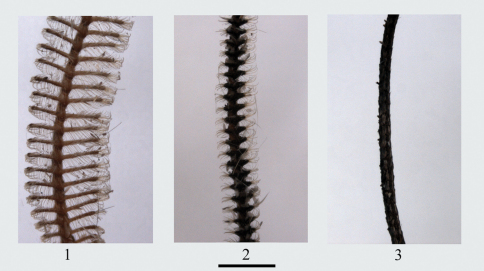
Antennae of *Biston*. **1** bipectinate, with long rami (male of *Biston melacron*) **2** bipectinate, with short rami (male of *Biston thibetaria*) **3** filiform (female of *Biston betularia*). Scale bar = 1 mm.

Head. Antennae bipectinate in male, rami short, moderately long or long, length tapering towards apex, often the distal part of antennae without rami; filiform in female (Figs 1–3). Frons not protruding, smooth-scaled. Tongue well developed. Labial palpus small, with hair-scales, not extending beyond frons. Compound eyes setose.

Thorax. Legs covered with hair-scales. Hind tibia slightly dilated, with two pairs of spurs in both sexes, without hair-pencil. Frenulum developed. Forewing without basal fovea in male, triangular, outer margin straight or waved, hindwing round, outer margin smooth, sometimes concave between M_1_ and M_3_ or protruding between M_1 _and CuA_1_. Wings white, pale yellow or greyish brown, transverse lines black, brown or white. Pattern of forewing: antemedial line slightly waved, often accompanied by a band basally; medial line waved, usually inconspicuous; postmedial line waved or dentate, sometimes protruding outwards between M_1_ and M_3_ and between CuA_2_ and 1A + 2A, often accompanied by a band posteriorly; submarginal line sometimes indistinct; terminal line sometimes appearing as a series of short stripes between veins; discal spot black or grey, shortly strip-like, dot-like or elliptic, pale-centred. Hindwing sometimes with basal line; medial line often indistinct, sometimes double; postmedial line waved or dentate, sometimes protruding outwards between M_1_ and M_3_; terminal line similar to those of forewing; discal spot sometimes smaller and less conspicuous than on forewing. Terminal spots occasionally present on both wings, wedge-shaped. Underside paler, transverse lines often similar to those of dorsal surface.

Venation. Forewing: Sc free, R_1_ and R_2_ usually stalked (separate in *Biston thoracicaria*), diverging before anterior angle of cell; R_2_ sometimes connected by a short transverse bar with R_3–4_ or R_3–5_; R_3–5_ before or from anterior angle of cell, not stalked with M_1_; M_1_ from anterior angle of cell; M_3_ from posterior angle of cell; CuA_1_ before posterior angle of cell. Hindwing: Sc+R_1_ close to cell less than onehalf length of cell; Rs before anterior angle of cell; M_1_ from anterior angle of cell; M_2_ absent; M_3_ from posterior angle of cell; CuA_1_ before or from posterior angle of cell; 3A absent.

Abdomen. Dorsum scattered with transverse lines or dots, sometimes with anal tuft. Third sternite of male abdomen without setal patch. Intersegmental membrane between abdomen and genitalia densely covered with elongate scales which partly developed to spines in a few species.

Male genitalia. Uncus short and broad, ratio of length to basal width variable, often bifurcate terminally, sometimes bifurcation very shallow or on ventral side below apex, so the latter apparently square or round. Arms of gnathos connected medially, with median process robust or slender, round, acute or square terminally. Valva simple; costa sclerotized, straight or incurved, with terminal half often broadened, bearing long setae from center to apex; sacculus sometimes sinuous. Saccus round or semicircular. Juxta well developed, pointed, or round or flat apically, sometimes elongate, without lateral brushes of long setae, except in *Biston melacron* Wehrli, 1941. Aedeagus often cylindrical, sclerotized dorsally; vesica usually wrinkled, scobinate, with or without cornuti; shape of cornuti various.

Female genitalia. Papillae anales covered with dense setae, occasionally elongate. Apophyses posteriores usually very long. Lamella postvaginalis sometimes present, oval or almost triangular. Ostium bursae occasionally weakly sclerotized. Ductus bursae striated longitudinally, sometimes sclerotized. Corpus bursae often long, membranous, sometimes curved medially, swollen anteriorly, often bearing a signum; signum elliptic, bar-like or irregularly shaped, often with marginal spines, sometimes weakly sclerotized around.

#### Diagnosis.

The genus *Biston* resembles *Cusiala* Moore and *Iulotrichia* Warren in: the postmedial lines of both wings often protrudes outwards between M_1_ and M_3_; the apex of the uncus is often bifurcated. But *Biston* differs from *Cusiala* and *Iulotrichia* in the following characters: the forewing fovea of the male is absent in *Biston* but present in *Cusiala* and *Iulotrichia*; in the male genitalia, the aedeagus vesica has numerous, very small, spine-like cornuti, arranged as two pair of longitudinal combs in *Cusiala* and *Iulotrichia*, which is absent in *Biston*. The members of *Biston* also resemble *Lycia* Hübner, 1825 and *Cochisea* Barnes & McDunnough, 1916, both of which belong to the former Bistonini. But both of these genera can be distinguished from *Biston* by the single pair of spurs on the hind tibia, as well as apterous or brachypterous female in *Lycia*, and absence of the tongue in *Cochisea*.

#### Distribution.

Holarctic, Oriental, and Ethiopian regions.

#### Biological notes.

The larva is often twig-like with the characteristic 45 degree resting posture and an obtusely cleft head (Holloway 1994). Singh (1953) recorded the larva of *Biston suppressaria* (Guenée, 1858). Issiki et al. (1977) illustrated the larva of *Biston robustum* Butler, 1879. Yamamoto et al. (1987) described and illustrated the larvae of *Biston betularia* (Linnaeus, 1758), *Biston robustum*, *Biston regalis* (Moore, 1888) and *Biston panterinaria*. Wagner (2001) recorded the larva of *Biston betularia*. Sato (2001) described the larva of *Biston marginata* Shiraki, 1913. Leong (2009) gave a description of the final instar larva and metamorphosis of *Biston pustulata*. Most species are highly polyphagous. The larval host plants have been recorded from the families Aceraceae, Adoxaceae, Anacardiaceae, Apocynaceae, Aquifoliaceae, Asteraceae, Berberidaceae, Betulaceae, Bombacaceae, Cannabaceae, Caprifoliaceae, Celastraceae, Compositae (Asteraceae), Cornaceae, Corylaceae, Cupressaceae, Elaeagnaceae, Ericaceae, Euphorbiaceae, Fagaceae, Ginkgoaceae, Grossulariaceae, Guttiferae (Clusiaceae), Iridaceae, Juglandaceae, Lardizabalaceae, Lauraceae, Leguminosae (Fabaceae), Lythraceae, Meliaceae, Melianthaceae, Myricaceae, Myrtaceae, Oleaceae, Palmae, Pinaceae, Platanaceae, Rhamnaceae, Rosaceae, Rutaceae, Salicaceae, Sapindaceae, Sterculiaceae, Styracaceae, Solanceae, Theaceae, Tiliaceae, Ulmaceae, Verbenaceae (summarized from Inoue 1965; Holloway 1994; Zhang 1994; Parsons et al. 1999; Sato 2001; Robinson et al. 2004). Patočka (2004) and Patočka and Turcani (2005) construct a key for the pupae of central European species. Nakamura (2004) described and gave a key for the pupae of Japanese species.

##### Species-group definitions based on morphology

**Group I:** *melacron*, *marginata*, *thoracicaria*, *betularia*, *robustum*, *regalis*, *mediolata*, *contectaria*, *bengaliaria*, *pustulata*, *suppressaria*.

Male antennae bipectinate, with long rami. Forewing outer margin straight, hindwing outer margin usually smooth, sometimes concave between M_1_ and M_3_. Postmedial lines of both wings protruding outwards between M_1_ and M_3_. Brown terminal spots absent from both wings. Patch of spines absent posterior to 8^th^ tergite on intersegmental membrane. Male genitalia: gnathos with median process acute or round terminally; terminal half of ventral margin of valva not protruding outwards; juxta elongate or not. Female genitalia: ovipositor with apophyses posteriores elongate.

**Group II**: *brevipennata*, *quercii*, *falcata*.

Male antennae bipectinate, with long rami. Forewing outer margin waved, hindwing outer margin protruding between M_3_ and CuA_1_. Postmedial lines of both wings not protruding outwards between M_1_ and M_3_. Brown terminal spots present on ends of forewing R_5_, M_1_, M_3_, CuA_1_, CuA_2_, hindwing Rs, M_1_, M_3_, CuA_1_, CuA_2_. Patch of spines absent posterior to 8^th^ tergite on intersegmental membrane. Male genitalia: gnathos median process round terminally; terminal half of ventral margin of valva protruding outwards; juxta elongate. Female genitalia: ovipositor with apophyses posteriores not elongate.

**Group III:** *perclara*, *thibetaria*, *panterinaria*.

Male antennaebipectinate, with short rami. Postmedial lines of both wings protruding outwards between M_1_ and M_3_. Brown terminal spots absent from both forewing and hindwing. Patch of spines present posterior to 8^th^ tergite on intersegmental membrane. Male genitalia: median process of gnathos round terminally; terminal half of ventral margin of valva protruding outwards; juxta not elongate. Female genitalia: ovipositor with apophyses posteriores elongate (Sato 1996).

##### Key to Chinese *Biston* species

**Table:** 

1	Outer margin of forewing waved	2
–	Outer margin of forewing not waved	4
2	Speckles on both wings dark brown	*Biston brevipennata*
–	Speckles on both wings black	3
3	Discal spots on hindwing distinct	*Biston quercii*
–	Discal spots on hindwing indistinct	*Biston falcata*
4	Male antennae bipectinate, with short rami	5
–	Male antennae bipectinate, with long rami	7
5	Discal spots on both wings indistinct	*Biston perclara*
–	Discal spots on both wings distinct	6
6	Discal spots on both wings black ringed and pale-centred	*Biston thibetaria*
–	Discal spots on both wings pale grey, round	*Biston panterinaria*
7	Antemedial line on forewing almost straight	8
–	Antemedial line on forewing waved	9
8	rojection between M_1_ and M_3_ of hindwing postmedial line round	*Biston mediolata* sp. n
–	Projection between M_1_ and M_3_ of hindwing postmedial line pointed	*Biston contectaria*
9	Forewing with R_1_ and R_2 _separate	*Biston thoracicaria*
–	Forewing with R_1_ and R_2 _stalked	10
10	Hindwing without basal line	11
–	Hindwing with basal line	14
11	Antennae totally bipectinate	*Biston robustum*
–	Antennae partially bipectinate	12
12	Projection between M_1_ and M_3_ of forewing postmedial line not bilobed	*Biston betularia*
–	Projection between M_1_ and M_3_ of forewing postmedial line bilobed	13
13	Outer margin of hindwing concave between M_1_ and M_3_	*Biston melacron*
–	Outer margin of hindwing not concave between M_1_ and M_3_	*Biston marginata*
14	Outer margin of hindwing concave between M_1_ and M_3_	*Biston regalis*
–	Outer margin of hindwing not concave between M_1_ and M_3_	15
15	Discal spots on hindwing black	*Biston bengaliaria*
–	Discal spots on hindwing pale grey	16
16	Apex of juxta evenly rounded in male genitalia	*Biston suppressaria*
–	Apex of juxta acute in male genitalia	*Biston pustulata*

### Biston melacron

Wehrli, 1941

http://species-id.net/wiki/Biston_melacron

Figs 4–6 70 97

Biston melacron Wehrli, 1941, *in* Seitz, *Gross-Schmett. Erde*, 4 (Suppl.): 430, pl. 35: h. Syntypes 3♂, China: West Tien-Mu-shan, 1600 m. (ZFMK)
Biston exotica Inoue, 1977, *Bull. Fac. domestic Sci., Otsuma Woman‘s Univ*., 13: 322, figs 65–67. Holotype ♂, Japan: Kochi Prefecture, Kubokawa. (BMNH) (Synonymized by Heppner and Inoue (1992))

#### Diagnosis.

The external characters of this species are close to those of *Biston marginata* as follows: the male antennae are partially bipectinate and filiform at tip; the forewing postmedial line bilobedly protrudes between M_1_ and M_3_, and slightly protrudes outwards between CuA_2 _and 1A + 2A. But it can be distinguished from *Biston marginata* by the following characters: the hindwing outer margin is concave between M_1_ and M_3_, whereas it is evenly round in *Biston marginata*; the transverse lines are black but dark brown in *Biston marginata*; the hindwing postmedial line is waved after M_3_, but straight in *Biston marginata*; the transverse lines on the underside of the wings are more conspicuous. The most distinct differences are in the male genitalia: the apex of the uncus is broader and bifurcated, whereas it is narrower and round in *Biston marginata*; the median process of the gnathos is broader and round terminally, while in *Biston marginata*, it is slenderer and acute apically; the setose area of the valva is much weaker; the juxta is narrower, and sharply pointed apically, while in *Biston marginata*, it is broader and round apically; the cornutus is shortly digitiform, but is thornlike in *Biston marginata*. In the female genitalia (Inoue 1977), the signum is much longer than in *Biston marginata*.

#### Material examineds.

**CHINA, Chekiang [Zhejiang]** (ZFMK): West Tien-Mu-shan, 1600 m, 27–29.IV.1932, coll. H. Höne, 3♂ (Syntypes); same data, 25.IV.1932, 1♂; same data, 25.V.1932, 1♂; same locality, 400 m, IV.1936, 1♂. **Fukien [Fujian]** (ZFMK):Kuatun, 2300 m, 3–7.IV.1938, 11.IV.1946, coll. J. Klapperich, 1♂. **Formosa **[**Taiwan]** (ZFMK): Puli, IV.1958, coll. ZSM, 1♂. **Sichuan** (IZCAS): Mt. Emei, Qingyinge, 800–1000 m, 17.IV–1.V.1957, coll. Zhu Fuxing & Huang Keren, 8♂.

#### Distribution.

China (Zhejiang, Jiangxi, Fujian, Taiwan, Sichuan), Japan, South Korea.

### Biston marginata

Shiraki, 1913

http://species-id.net/wiki/Biston_marginata

Figs 7–10 71 98 117

Biston marginata  Shiraki, 1913, *Spec. Rep. Formosa agric. Exp. Stn*, [Special reports No. 8] Publication no. 68: 433, pl. 44. Syntypes, China: Taiwan.
Biston fragilis Inoue, 1958, *Tinea*, 4 (2): 254, pl. 34, fig. 30. Holotype ♂, Japan: Oita Prefecture, Saeki. (BMNH) (Synonymized by Inoue (1965))

#### Diagnosis.

The diagnostic characters of external morphology of the species can be seen in the previous species. The male genitalia of the species are close to those of *Biston suppressaria*. But it can be distinguished from the latter by the following characters: the vesica is less strongly sclerotized posteriorly; the cornutus is small and spine-like but absent in *Biston suppressaria*. The female genitalia are similar to those of *Biston betularia*, but they differ in the following characters: the ductus bursae is shorter and the antrum is absent; the corpus bursae is almost even in width, while in *Biston betularia* it is enlarged, wrinkled and weakly sclerotized posteriorly, narrow medially and swollen anteriorly; the signum is oval with several marginal spines, but a transverse bar in *Biston betularia*.

#### Material examineds.

**CHINA, Fukien [Fujian]** (ZFMK): Kuatun, 2300 m, 3.IV.1938, coll. J. Klapperich, 1♂. **Formosa **[**Taiwan]** (ZFMK): Nantou, SW Tsuifeng, 2100m, 16.III.1996, coll. Csoevari and Steger, 2♂; Puli, IV.1958, coll. ZSM, 2♂. **Hunan **(IZCAS): Dongan, 24.II.1955, 4♀; Changning, 1981, 1♀. **Guangdong **(IZCAS): Guangzhou, VIII.1984, 1♂; Yingde, Chayesuo, 1♂. **Guangxi **(IZCAS): Bobai, Yunfei, Fenchang, 2–3.I.1986, coll. Wang Jijian, 1♂2♀. **Chongqing **(IZCAS): Beipei, Jinyunshan, 10–12.II.1987, 1♂1♀. **Yunnan **(IZCAS): Suining, VII.1980, 1♂. **VIETNAM** (ZFMK): Tam Dao, 950 m and Fan-si-pan, 1520 m, III.1995, large series of males and a few females.

#### Distribution.

China (Zhejiang, Jiangxi, Hunan, Fujian, Taiwan, Guangdong, Guangxi, Chongqing, Yunnan), Japan, Vietnam.

**Figures 4–20.**
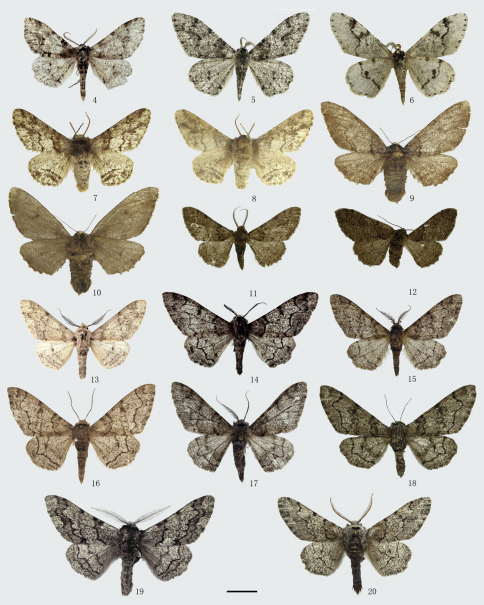
Adults of *Biston*. **4**–**6** *Biston melacron*. **4** male (holotype **5** male **6** ditto, underside **7**–**10** *Biston marginata*. **7** male **8** ditto, underside **9** female **10** ditto, underside **11**–**12** *Biston thoracicaria*. **11** male **12** female **13**–**16** *Biston betularia parva*. **13** male (holotype of *Biston huberaria tienschana*) **14** male (holotype of *Biston cognataria sinitibetica*) **15** mal **16** female **17**–**18** *Biston betularia nepalensis*. **17** male **18** female **19**–**20** *Biston robustum*. **19** male (holotype of *Biston robustum kiangsua*) **20** male. Scale bar = 1 cm.

### Biston thoracicaria

(Oberthür, 1884)

http://species-id.net/wiki/Biston_thoracicaria

Figs 11, 12 72 99 118

Jankowskia thoracicaria Oberthür, 1884, *Études ent.*, 9: 26, pl. 2, fig. 8. 4 Syntypes 4♂♀, Russia: Sidemi. (ZFMK)
Lycia tortuosa Wileman, 1911, *Trans. ent. Soc. Lond.*, 1911 (2): 310, pl. 30, fig. 1, pl. 31, fig. 27. Holotype ♂, Japan: Oshima, Tobetsu. (BMNH) (Synonymized by Inoue, (1976))
Biston thoracicaria : Prout, 1915, *in *Seitz, *Macrolepid. World*, 4: 359, pl. 19: g.

#### Diagnosis.

The external characters of this species are close to those of *Biston betularia* as follows: the male antennae are partially bipectinate and filiform at apex; the forewing postmedial line protrudes outwards between M_1_ and M_3_ and between CuA_2 _and 1A + 2A; the discal spots of both wings are stripe-like. But it can be distinguished from *Biston betularia* by the following characters: distinctly smaller; the wing colour is dark brown, but greyish black in *Biston betularia*; the hindwing basal line is present, but absent in *Biston betularia*; the forewing R_1_ and R_2_ are separate, but stalked in *Biston betularia*. In the male genitalia, the apex of the uncus and the median process of the gnathos are more slender than those of *Biston betularia*; the valva is more slender and longer; the juxta is much narrower; the cornutus is small and spine-like, whereas *Biston betularia* has two kinds of cornuti, one is a large bundle of spines, the other is a small tuft of spines. In the female genitalia, the corpus bursae is curved in the anterior half, while in *Biston betularia*, it is expanded posteriorly and narrow medially; the signum is elliptic, with several small marginal spines, whereas it is bar-like and without marginal spines in *Biston betularia*.

#### Material examineds.

**RUSSIA** (ZFMK): Sidemi, coll. Oberthür, 2♂ (Syntypes); Dairen, Mantschourie, coll. H. Höne, 1♂ (Syntype). **CHINA, Shaanxi** (ZFMK): Tsinling, Tapaishan, coll. H. Höne, 8♂1♀. **Shantung [Shandong]** (ZFMK): Tai-Shan, 1550 m, coll. H. Höne, large seriesincluding 3♀. **Jiangsu** (ZFMK): Nanking, Lungtan, coll. H. Höne, 3♂; **Zhejiang** (ZFMK): East-tien-mu-shan, coll. H. Höne, 3♂; **Yuennan** **[Yunnan]** (ZFMK): Li-kiang, coll. H. Höne,1♂. **Beijing** (IZCAS): 5.VII.1949, 1♀; VII.1972, coll. Zhang Baolin, 3♂; Baihuashan, 9.VII.1973, coll. Han Yinheng, 1♀. **Hebei** (IZCAS): Yibao, 12.VIII.1972, coll. Zhang Baolin, 3♂. **Henan** (IZCAS): Xinyang, Jigongshan, 250 m, 20–21.VII.2002, coll. Han Hongxiang, 1♂. **Shaanxi** (IZCAS): Huangbaiyuan, 13.VII.1980, coll. Zhang Baolin, 1♂. **Gansu** (IZCAS): Wenxian, Bikou, 720 m, 28.VII.1999, coll. Yao Jian, 1♂. **Zhejiang** (IZCAS): Tianmushan, 4.V.1980, coll. Cai Rongquan, 1♂. **Hubei** (IZCAS): Shennongjia, Shouge, 900 m, 18.VI.1981, coll. Han Yinheng, 2♂.

#### Distribution.

China (Beijing, Hebei, Shandong, Henan, Shaanxi, Gansu, Jiangsu, Zhejiang, Hubei, Yunnan), Russia, Japan, North Korea, South Korea.

### Biston betularia

(Linnaeus, 1758)

http://species-id.net/wiki/Biston_betularia

Figs 13–18 73, 74 100, 101 119, 120

Phalaena(Geometra) betularia Linnaeus, 1758, *Syst. Nat*., (Ed. 10) 1: 521. Syntype(s), Europe. (LSL)
Phalaena (Noctua) p-graecum Poda, 1761, *Insecta Musei Graecensis*: 90. Syntype(s). (Synonymized by Wehrli (1941))
? marmoraria Sepp, 1792, *Nederlandsche Insecten*, 2: pl. 10, pl. 11, Syntype(s), Netherlands. (Treated as a synonym of *Biston betularia betularia* by Parsons et al. (1999))
Phalaena (Geometra) ulmaria Borkhausen, 1794, *Natur. eur. Schmett*., 5: 181. Syntype(s), Europe. (Treated as a synonym of *Biston betularia betularia* by Parsons et al. (1999))
Eubyja betularia : Hübner, 1825, *Verz. bekannter Schmett*.: 318.
Amphidasis huberaria Ballion, 1866, *Horae Soc. ent. ross*., 4: 29, pl. 1, fig. 1. Syntype(s), Russia: Western Siberia, near Omsk. (ZISP) (Synonymized by Vijdalepp, (1979))
Amphidasys betularia var. doubledayaria Millière, 1870, *Iconogr. Descr. Chenilles Lépid. inédits*, 3: 117, pl. 111, fig. 1. Syntypes, including at least 3♂, 4♀, England. (Synonymized by Wehrli (1941))
Eurbyjodonta concinna Warren, 1899, *Novit. zool*., 6: 50. Holotype ♂, Kazakhstan?: Ili district. (BMNH) (Synonymized by Wehrli (1941))
Biston betularia : Prout, 1915, *in *Seitz, *Macrolepid. World*, 4: 358, pl. 19: g.
Biston cognataria alexandrina Wehrli, 1941, *in* Seitz, *Gross-Schmett. Erde*, 4 (Suppl.): 432, pl. 36: a. Syntypes 4♂, Kirghizstan: Alexander Mountains. (ZFMK) (Treated as a synonym of *Biston betularia betularia* by Parsons et al. (1999))
Biston (Eubyjodonta) huberaria tienschana Wehrli, 1941, *in* Seitz, *Gross-Schmett. Erde*, 4 (Suppl.): 435, pl. 36: d, g. Holotype ♂, China: Xinjiang, Ürümqi, Tian-shan. (ZFMK) (Treated as a synonym of *Biston huberaria* by Parsons et al. (1999))
Biston cognataria sinitibetica Wehrli, 1941, *in* Seitz, *Gross-Schmett. Erde*, 4 (Suppl.): 433, pl. 36: a. Syntypes ♂, ♀, China (west): Kangding. (ZFMK) (Treated as a synonym of *Biston betularia parva* Leech, 1897 by Parsons et al. (1999))

#### Diagnosis.

See the previous species.

#### Material examineds.

**CHINA, Xinjiang **(ZFMK): Urumtschi, Tian-schan**, **1♂ (Holotype of *Biston huberaria tienschana*). **Szetschwan [Sichuan]** (ZFMK): Tachien-lu [Kangding], 1♂ (Holotype of *Biston cognataria sinitibetica* Wehrli, 1941). **Heilongjiang**(IZCAS): Dailing, 390 m, 3–9.VII.1962, coll. Bai Jiuwei, 3♂; Jiamusi, 23.VIII.1970, coll. Song Shimei, 1♂; Wuchang, 8–10.VII.1970, 2♂2♀. **Jilin** (IZCAS): Changbaishan, 2–13.VII.1982, coll. Zhang Baolin, 10♂2♀; Beipiao, 1984, coll. Liu Jin, 1♂. **Inner Mongolia** (IZCAS): Wuchagou, 21.VII.1981, 1♂; Jiwen, 15–27.VII.1982, 1♂1♀; Genhe, 4.VII.1983, 1♂1♀; Xilin Gol, 1100 m, 21.VII.1985, coll. Liu Dajun, 2♂; Oragxan, 4.VII.1983, 1♀; Oragxan, 27.VI.1985, coll. Xue Dayong, 1♂; Chen Barag, Bayan Hure, 2–3.VII.1986, 1♂1♀; Oroqen, Alihe, 31.VII.1986, coll. Gong Yushan, 1♂; Dayangshu, 20–23.VI.1983, coll. Xue Dayong, 2♂1♀. **Beijing** (IZCAS): Sanpu, 16.VII.1965, 1♂; Baihuashan, 16.VII–1.VIII.1972, coll. Han Yinheng and Zhang Baolin, 8♂; Mentougou, Xiaolongmen, 1100 m, 23.VI.2001, coll. Xue Dayong, 1♂; Mentougou, Liyuanling, 1100 m, 11–12.VIII.2004, coll. Li Hongmei, 1♂. **Hebei** (IZCAS): Chengde, 11.VI.1980, 2♂; Chicheng, Longmensuo, Liuzhuangzi, 10–11.VIII.2006, coll. Yang Chao, 1♂1♀. **Shanxi** (IZCAS):Yuncheng, Zhongtiaoshan, coll. Cao Tianwen, 4♂. **Henan** (IZCAS): Linxian, 1981, 1♂; Lingbao, Chuankou, 20.VII.1981, 1♂. **Shaanxi** (IZCAS): Huangbaiyuan, 13.VII.1980, coll. Zhang Baolin, 1♂; Yan’an, 5.VI.1981, 3♂2♀; Taibai, 3.VII.1981, 1♀; Liuba, 1020 m, 18.VII.1998, coll. Zhang Xuezhong, 2♂. **Ningxia** (IZCAS): Jingyuan, 1998–2295 m, 25.VI.–10.VII.2008, coll. Song Wenhui, 32♂1♀; Longde, 2165–2330 m, 3–5.VII.2008, coll. Song Wenhui, 11♂2♀. **Gansu** (IZCAS): Yongdeng, Liancheng Linchang, 20.VII–15.VIII.1985, coll. Meng Feng, 2♂; Yongdeng, Tulugou, 2280 m, 25–29.VII.1991, coll. Xue Dayong, 1♂3♀; Yongdeng, Liancheng Linchang, Tulugou, 2600 m, 8.VIII.2005, coll. Li Jing and Han Hongxiang, 2♂; Zhuoni, 2500 m, 4.IX.1990, coll. Xue Dayong, 1♂; Kangxian, Baiyunshan, 1250–1750 m, 12.VII.1998, coll. Yao Jian, 1♂; Zhouqu, Shatan Linchang, 2350 m, 4–5.VII.1998, coll. Wang Hongjian and Wang Shuyong, 18♂2♀; Zhouqu, Shatan Linchang, 2357 m, 22.VIII.2001, coll. Cao Xiuwen, 1♂; Diebu, Anzigou, 20.VI–20.VII.2000, 4♂; Dangchang, 1800 m, 7.VII.1998, coll. Yao Jian, 3♂; Wenxian, Qiujiaba, 2350 m, 7–22.VII.1999, coll. Yao Jian, 3♂1♀; Wenxian, Tielou, 1450 m, 24.VII.1999, coll. Zhu Chaodong, 8♂. **Qinghai** (IZCAS): Menyuan, Xianmi, 2800 m, 23–24.VII.1992, coll. Xue Dayong, 1♂5♀; Huanglin, 17.VII.1977, coll. Zhang Baolin, 1♂; Huzhu, Beishan, 2300 m, 30.VII.1991, coll. Xue Dayong, 1♂; Huzhu, Beishan Linchang, Langshidang, 2600 m, 6–7.VIII.2005, coll. Xue Dayong, 4♂3♀; Tongren, Maixiu, 2950 m, 30.VII–1.VIII.1992, coll. Xue Dayong, 14♂2♀; Xining, 6.VII.1981, 1♀. **Xinjiang** (IZCAS): Ürümqi, 21.VI.1965, 2♂; Ürümqi, 1974.VI.22, coll. Chen Yixin, 1♂; Aksu, 19.VII.1982, 1♂. **Fujian** (IZCAS): Mt. Wuyi, Sangang, 5–9.VI.1983, coll. Wang Linyao, 4♂. **Sichuan** (IZCAS): Batang, 1974, 8♂; Mt. Emei, Qingyinge, 800–1000 m, 1.V.1957, coll. Huang Keren, 1♂; Kangding, 30.VI–6.VII.1979, coll. Chen Tailu, 8♂1♀. **Yunnan** (IZCAS): Deqin, 3250 m, 15.VII.1982, coll. Wang Shuyong, 2♂; Xiaozhongdian, 3100 m, 31.VII.1984, coll. Chen Yixin, 1♀; Lijiang, Shiyan Linchang, 2460 m, V.1979, 1♂; Lijiang, Yulongshan, 2700–2850 m, 17–27.VII.1984, coll. Chen Yixin and Liu Dayun, 4♂1♀; Lijiang, Yuhu, 2700 m, 27.VII.1984, coll. Liu Dayun, 1♂; Lijiang, Gaoshan Zhiwuyuan, 3260 m, 15–18.VI.2009, coll. Qi Feng, 1♂; Cuishan, Shatanshao, 2300 m, 13.VIII.1980, 1♂; Kunming, Jinzhusi, 1880 m, 15.VIII.1980, 1♂; Yongping, Beidou, Qianmatang, 2200 m, 10.VIII.1980, 1♂. **Tibet** (IZCAS): Qamdo, 19.VIII.1984, coll. Jiang Basang, 1♀; Markam, Pula, 23.VII.1984, coll. Jiang Basang, 2♂; Nyingchi, Shang Zayü, 1960 m, 21–23.VIII.2005, coll. Wang Xuejian, 6♂; Nyingchi, Bayi, 2999 m, 1–3.VIII.2006, coll. Lang Songyun**, **2♀; Mainling, Pai, Zhuanyunzhan, 2883 m, 4–6.VIII.2006, coll. Lang Songyun, 1♀; Yadong, 2800 m, 8.VI.1961, coll. Wang Linyao, 1♂; Yadong, 14.VI.1983, coll. Wangjia Tsering, 1♀. **MONGOLIA** (IZCAS): Batshireet, Hentiy, 1108 m, 29.VI.2009, coll. Chen Fuqiang, 20♂; Binder, Henity, 1032 m, 29.VI–1.VII.2009, coll. Chen Fuqiang, 20♂5♀; Dadal, Hentiy, 944 m, 2.VII.2009, coll. Chen Fuqiang, 10♂; Choybalsan, Dornod, 733 m, 6.VII.2009, coll. Chen Fuqiang, 2♂. More material from Shanxi, Shaanxi, Sichuan, and Yunnan in coll. ZFMK.

#### Distribution.

China (Heilongjiang, Jilin, Inner Mongolia, Beijing, Hebei, Shanxi, Shandong, Henan, Shaanxi, Ningxia, Gansu, Qinghai, Xinjiang, Fujian, Sichuan, Yunnan, Tibet), Russia, Mongolia, Japan, North Korea, South Korea, Nepal, Kazakhstan, Kirghizstan, Turkmenistan, Georgia, Azerbaijan, Armenia, Europe, North America.

#### Remarks.

There are two subspecies of *Biston betularia* distributed in China, they are *Biston betularia parva* Leech, 1897 (*Biston robustum* var. *parva* Leech, 1897, *Ann. Mag. nat. Hist.*, (6) 19: 323. Syntypes 1♂, 2♀, China: Kangding. (BMNH)) (Figs 13–16, 73, 100, 119) and *Biston betularia nepalensis* Inoue, 1982 (*Biston betularia nepalensis* Inoue, 1982, *Bull. Fac. domest. Sci. Otsuma Wom. Univ.*, 18: 175, figs 40a, b. Holotype ♂, Nepal: Tukcha, near Daulagiri. (BMNH)) (Figs 17–18, 74, 101, 120). In China, the former is widely distributed in the greater part of the country, the latter is distributed in Yunnan and Tibet, and can be distinguished from the former by the weaker transverse lines and the blunter projections of the postmedian lines on both wings.

### Biston robustum

Butler, 1879

http://species-id.net/wiki/Biston_robustum

Figs 19, 20 75 102

Biston robustum Butler, 1879, *Ann. Mag. nat. Hist.*, (5) 4: 371. Syntype(s), Japan: Yokohama. (BMNH)
Biston robustum kiangsua Wehrli, 1941, *in* Seitz, *Gross-Schmett. Erde*, 4 (Suppl.): 433, pl. 36: b. Holotype ♂, China: Shanghai. (ZFMK) (Treated as a synonym of *Biston robustum robustum* by Parsons et al. (1999))

#### Diagnosis.

The external characters of this species are close to those of *Biston porphyria* (Butler, 1889) (India) as follows: the male antennae are bipectinate to tip; greyish brown bands are present basally of the antemedial line of the forewing and distally of the postmedial lines of both wings; the forewing medial line converges with the postmedial line at 1A + 2A; the hindwing postmedial line acutely protrudes between M_1_ and M_3_; the submarginal lines of both wings are dark grey. But the species can be distinguished by the following characters: this species (length of forewing: 28–30 mm in male) is larger than *Biston porphyria*; the wings are broader; the hindwing medial line is more conspicuous. The male genitalia of the species are similar to those of *Biston betularia* as follows: the apex of the uncus is bifurcated; the median process of the gnathos is about one-half length of the uncus; the juxta is long, narrow, and acute apically. But it can be distinguished from *Biston betularia* by the longer and narrower valva and the absence of cornuti.

#### Material examineds.

**CHINA, Shanghai** (ZFMK): 1♂ (Holotype of *Biston robustum kiangsua*). **Shandong** (IZCAS): Gujiding, 13.IV.1981, 3♂. **Jiangsu** (IZCAS): Nanjing, Qixiaqu, Yaohuamen, 16.III.2006, coll. Lang Songyun, 3♂. More material from Shaanxi, 2♂ from Taiwan, many from Japan, 2♂ from Korea and many from Vietnam in coll. ZFMK.

#### Distribution.

China (Shandong, Shaanxi, Shanghai, Jiangsu, Jiangxi, Taiwan), Japan, Russia, North Korea, South Korea, Vietnam.

#### Remarks.

There are two Chinese subspecies of *Biston robustum*, they are *Biston robustum robustum* Butler, 1879 and *Biston robustum subrobustum* Inoue, 1964 (*Biston robustum subrobustum* Inoue, 1964, *Kontyû*, 32 (2): 338, pl. 8, fig. 3. Holotype ♂, Taiwan (central): Puli. (BMNH)). The former is distributed in the mainland China, the latter is distributed in Taiwan. The description of *Biston robustum kiangsua* Wehrli was based on a single, rather aberrant specimen, with a single printed label “Shanghai China”. Similar forms occur in Japan, as mentioned by Wehrli (1941). So this name very probably does not denote a valid subspecies and it is synonymized correctly.

### Biston mediolata sp. n.

urn:lsid:zoobank.org:act:3657C566-2007-4007-B07E-1C3606EF048D

http://species-id.net/wiki/Biston_mediolata

**Figures 21–30.**
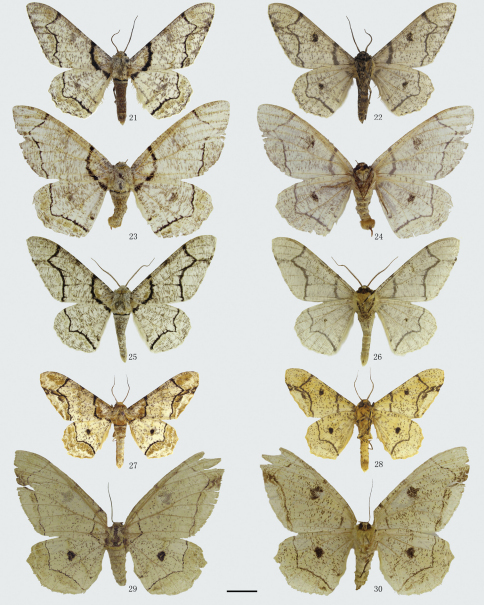
Adults of *Biston*. **21**–**24** *Biston mediolata*sp. n. **21** male (holotype) **22** ditto, underside **23** female (paratype) **24** ditto, underside. **25**–**26** *Biston contectaria*. **25** male **26** ditto, underside; **27**–**30** *Biston bengaliaria*. **27** male **28** ditto, underside **29** female **30** ditto, underside. Scale bar = 1 cm.

Figs 21–24 76 103 121

Biston contectaria Walker, sensu Xue, 1992a; *in* Liu, *Iconography of Forest Insects in Hunan China*: 880.

#### Description.

##### Head.

Antennae about one-third length of forewing, bipectinate in basal three-fifths, filiform in terminal two-fifths, rami short, length of longest ramus about the same as diameter of antennal shaft; filiform in female. Frons not protruding, smooth-scaled, with basal half white, upper half black. Labial palpus black, small, not extending beyond frons. Vertex white.

Thorax. Dorsum greyish white, scattered with black scales. Patagia and tegulae greyish white, mottled with black scales, pale yellow distally. Posterior margin of metanotum with yellow scales medially. Hind tibia with two pairs of spurs in male, slightly dilated, without hair-pencil. Forewing length: male 32–34 mm; female 42 mm. Forewing outer margin almost straight, hindwing round. Wings white, with pale grey striation. Pattern of forewing: antemedial line black, almost straight anteriorly, distinct, accompanied by a pale yellow band basally; medial line greyish yellow, indistinct; postmedial line black, distinct, acutely protruding outwards between M_1_ and M_3_, shallowly protruding outwards between CuA_2_ and 1A + 2A, sometimes internally dentate on veins; a pale yellow band distally of postmedial line, diffused with black patches; terminal line black; fringe pale yellow mixed with black; discal spot present as pale grey dot, indistinct. Hindwing with basal line black, distinct; medial line only distinct near anal margin; postmedial line black, bluntly protruding outwards between M_1_ and M_3_, sometimes appearing as black serrations on veins; a pale yellow band distally of postmedial line; terminal line indistinct; fringe and discal spot similar to those of forewing. Underside white; transverse lines dark grey, similar to those of forewing, discal spot black, heavy, on hindwing smaller than on forewing.

##### Abdomen.

First abdominal segment greyish white with black basal margin, remaining segments yellowish brown, scattered with black dots. Setal patch absent on third sternite of male abdomen.

##### Male genitalia.

Uncus somewhat trapeziform, about three-fifths length of basal width. Gnathos with median process short and broad, round terminally, about two-fifths length of uncus. Valva simple, broad at base, gradually narrowing apically, about twice as long as basal width; costa sclerotized, incurved medially, expanded with particularly dense setae basally. Saccus round, about one-third length of basal width. Juxta short and broad, almost round. Coremata not developed. Aedeagus cylindrical, sclerotized dorsally; vesica scobinate, cornutus shaped as a narrow band.

##### Female genitalia.

Ovipositor with apophyses posteriores elongate. Lamella postvaginalis small and almost triangular. Ostium bursae sclerotized. Ductus bursae striated longitudinally, short. Corpus bursae long, curved medially, swollen anteriorly, bearing a signum; signum long elliptic with marginal spines.

##### Diagnosis.

The wing pattern of this species is similar to that of *Biston contectaria* as follows: the forewing outer margin is almost straight anteriorly; the antemedial line is black, broad and almost straight; the postmedial line acutely protrudes between M_1_ and M_3_; the medial lines of both wings are greyish yellow and indistinct; pale yellow bands are present basally of the forewing antemedial line and distally of the postmedial line of both wings; the hindwing basal line is black and distinct. But this species is smaller and can be distinguished by the following characters: the postmedial lines of both wings are narrower; the forewing postmedial line weakly protrudes outwards between CuA_2 _and 1A + 2A, while in *Biston contectaria*, it is straight; the protrusion between M_1_ and M_3_ of the hindwing postmedial line is round but sharply angled in *Biston contectaria*; the discal spots on the underside of both wings are larger and heavier. In the male genitalia: the much broader uncus and valva of the new species are distinctly different from *Biston contectaria*. The female genitalia are similar to those of *Biston panterinaria* as follows: the apophyses posteriores are long; the ostium bursae is sclerotized; the ductus bursae is very short; the corpus bursae is curved medially; the signum is elliptic and narrow. But it can be distinguished from *Biston panterinaria* by presence of the lamella postvaginalis.

#### Type material.

Holotype(IZCAS), ♂, **CHINA, Hubei:** Xingshan, Longmenhe, 1200 m, 18.VII.1993, coll. Song Shimei. Paratypes, **CHINA, Shaanxi**(IZCAS): Liuba, Miaotaizi, 1470–1550 m, 1–2.VII.1999, coll. He Tongli, 2♂. **Gansu**(IZCAS): Kangxian, Qinghe Linchang, 1450–1650 m, 15.VII.1998, coll. Yao Jian, 1♂; Wenxian, Dianba, 23.VI.1992, coll. Wang Hongjian, 1♂. **Hubei**(IZCAS):Xingshan, Longmenhe, 1260–1350 m, 17–23.VI., 14–21.VII.1993, coll. Huang Runzhi, Song Shimei and Yao Jian, 11♂; Hefeng, 1240 m. 21–31.VII.1989, coll. Li Wei, 4♂; Hefeng, Fenshuiling, 1240 m, 1989.VII.29, coll. Li Wei, 1♀; Badong, 19.V.1989, coll. Li Wei, 1♂. **Hunan**(IZCAS): Chenzhou, Guanlisuo, 8.VII.1969, 1♂. **Fujian**(IZCAS): Mt. Wuyi, Sangang, 740 m, 25.V.–30.VI.1960, coll. Zhang Yiran, 2♂; Mt. Wuyi, Sangang, 10.V.1981, coll. Wang Jiashe, 1♂; Mt. Wuyi, Sangang, 14.VI.1983, coll. Wang Linyao, 1♂. **Guangxi **(IZCAS):Nanningqu, Linkesuo, 110 m, 17.IV.1984, 1♂; Mao’ershan, 1600 m, 15.VII.1985, coll. Fang Chenglai, 1♂; Jinxiu Shengtangshan, 900–1900 m, 29.VI.2000, coll. Li Wenzhu, 1♂; Jinxiu, Linhai, Shanzhuang, 1000 m, 2.VII.2000, coll. Li Wenzhu, 3♂. **Sichuan**(IZCAS):Mt. Emei, Qingyinge, 800–1000 m, 22.V., 21–26.VI., 11–16.VII.1957, coll. Huang Keren and Zhu Fuxing, 7♂. **Hubei** (ZFMK): Wufeng, Yizhuxiang, 1560 m, VI.1998, coll. Wang and Li 1♂. **Fukien** **[Fujian]** (ZFMK): Kuatun, 2300 m, 28.V., 1.VI.1938, coll. J. Klapperich, 2♂. **Hainan** (ZFMK): Wuzhishan, 1600 m, VII.1998, coll. Yin and Wang, 6♂. **VIETNAM** (ZFMK): Mt. Fan-si-pan, 1600–1800 m, 8-29.V.1993, coll. Sinjaev and Simonov, 2♂.

#### Etymology.

The specific name is from the Latin prefix *medio*- and the word *latus*, which means medially and broad, refers to the shape of the valva.

#### Distribution.

China (Shaanxi, Gansu, Hubei, Hunan, Fujian, Hainan, Guangxi, Sichuan), Vietnam.

### Biston contectaria

(Walker, 1863)

http://species-id.net/wiki/Biston_contectaria

Figs 25, 26 77 104

Amphidasis contectaria Walker, 1863, *List Specimens lepid. Insects Colln Br. Mus*., 26: 1529. Holotype ♀, India. (BMNH)
Biston (*Cusiala*) *bengaliaria* f. *contectaria*: Hampson, 1895, *Fauna Br. India* (Moths), 3: 248.
Biston contectaria : Yazaki, 1992, *in* Haruta, *Tinea*, 13 (Suppl. 2): 33.

#### Diagnosis.

The external characters of this species are close to those of *Biston suppressaria* and *Biston inouei* Holloway, 1994 (Borneo), but it can be distinguished from those species by the following differences: this species (length of forewing: 27–28 mm in male) is larger than *Biston suppressaria* and smaller than *Biston inouei*; the protrusion between M_1_ and M_3_ of the forewing postmedial line is relatively acute, but blunt or bilobed in *Biston suppressaria* and *Biston inouei*; the hindwing basal line is more distinct in *Biston contectaria* and *Biston inouei*; the projection between M_1_ and M_3_ of the the hindwing postmedial line is relatively acute in *Biston contectaria* and *Biston suppressaria*, but blunt in *Biston inouei*. In the male genitalia, the apex of the uncus is broader than that of *Biston suppressaria* and *Biston inouei*, and is almost not bifurcated; the median process of the gnathos is shorter and round distally, whereas it is longer and pointed in *Biston suppressaria* and *Biston inouei*; the costa and the ventral margin of the valva are curved, while those of *Biston suppressaria* and *Biston inouei* are less curved or even incurved or concavely curved; the costa is expanded and has dense setae basally, while it is straight in *Biston suppressaria* and *Biston inouei*; the juxta is shorter and less pointed apically.

#### Material examineds.

**CHINA, Yunnan** (IZCAS):Yunxian, Xingfuzhen, 103 m, 2.VIII.1980, coll. Yang Tianshou, 1♂; Tengchong, Dahaoping, 2020 m, 24–26.V.1992, coll. Xue Dayong, 6♂; Tengchong, Heinitang, 1930 m, 28–30.V.1992, coll. Xue Dayong, 1♂; Dulongjiang, 1500 m, 29.V.2006, coll. Xiao Ningnian, 1♂. More material from several parts of Yunnan in coll. ZFMK.

#### Distribution.

China (Yunnan), India, Nepal.

### Biston bengaliaria

(Guenée, 1858)

http://species-id.net/wiki/Biston_bengaliaria

Figs 27–30 78 105 122

Amphidasis bengaliaria Guenée, 1858, *in* Boisduval & Guenée, *Hist. nat. Insectes* (Spec. gén. Lépid.), 9: 210; *ibidem* (1858) Atlas; pl. 4, fig. 2. Syntypes 3♂, 1♀, Bengal; India (central). (BMNH)
Blepharoctenia bengaliaria : Warren, 1894, *Novit. zool*., 1: 428.
Biston (Cusiala) bengaliaria : Hampson, 1895, *Fauna Br. India* (Moths), 3: 247.
Biston bengaliaria : Yazaki, 1992, *in* Haruta, *Tinea*, 13 (Suppl. 2): 33.

#### Diagnosis.

The external characters of this species are close to those of *Biston contectaria*, but it can be distinguished from that species by the following differences: the wings are pale yellow but white in *Biston contectaria*; the forewing postmedial line is much narrower and protruding outwards between CuA_2 _and 1A + 2A, while in *Biston contectaria*, it is broader and without such a protrusion; the discal spot on the hindwing upperside is large, round, black, while in *Biston contectaria* it is almost absent; the discal spots on the underside of both wings are larger and heavier. The male genitalia are close to those of *Biston suppressaria*, but it can be distinguished by the square apex of the juxta, the shorter median process of the gnathos and the presence of a cornutus which is a short spinous patch. The female genitalia are similar to those of *Biston suppressaria*. But it differs in that the corpus bursae is coiled anteriorly; the signum is longer and narrower; the ostium bursae is more strongly sclerotized.

#### Material examineds.

**CHINA, Yunnan** (IZCAS): Yuanjiang, 500 m, 12.V.1957, coll. Liang Qiuzhen, 1♀; Cangyuan, 750 m, 22.V.1980, coll. Song Shimei, 1♂; Xishuangbanna, Menglun, 27.V.1964, coll. Zhang Baolin, 1♂; Xishuangbanna, Bubang, 700 m, 14.IX.1993, coll. Cheng Xinyue, 3♂. **Tibet** (IZCAS): Mêdog, Beibung, 700–800 m, 11.VI.1983, coll. Lin Zai, 2♂; Mêdog, Yarang, 902–1091 m, 14–23.VIII.2006, coll. Lang Songyun, 6♂. More material from several parts of Yunnan in coll. ZFMK.

#### Distribution.

China (Yunnan, Tibet), India, Bengal, Thailand.

### Biston pustulata

(Warren, 1896) New to the fauna of China

http://species-id.net/wiki/Biston_pustulata

Figs 31, 32 79 106

Buzura pustulata Warren, 1896, *Novit. zool*., 3: 401. Holotype ♂, Peninsular Malaysia: Perak. (BMNH)
Biston pustulata : Holloway, 1994, *Malay. Nat. J.*,47: 210, pl. 12, fig. 452.

#### Description.

Head. Male antennae about two-fifths length of forewing, bipectinate in basal two-thirds, filiform in terminal one-third, rami long, length of longest ramus about three and half times diameter of antennal shaft. Frons not protruding, smoothscaled, with basal half pale yellow, upper half black. Labial palpus black, small, pale yellow apically, not extending beyond frons. Vertex pale yellow.

Thorax. Dorsum white-dotted with black scales. Patagia and tegulae white, mottled with black scales, yellow apically. Posterior margin of metanotum with two pairs of pale yellow spots. Hind tibia with two pairs of spurs in male, slightly dilated, without hair-pencil. Forewing length: male 29 mm. Forewing outer margin straight, hindwing round. Wings greyish white, dotted with pale grey scales. Pattern of forewing: antemedial line black, slightly waved, distinct, accompanied by a pale yellow band basally; medial line pale yellow, indistinct; postmedial line black, distinct, bilobedly protruding outwards between M_1_ and M_3_, then incurved, protruding outwards between CuA_2_ and 1A + 2A; a pale yellow band distally of postmedial line; black patches present between M_1_ and CuA_1_ distally of postmedial line and reaching outer margin between M_1_ and M_3_; submarginal line white, dentate; terminal line a series of short black strips between veins; fringe yellow mixed with black; discal spot present as grey dot. Hindwing with basal line black; medial line pale yellow, indistinct; postmedial line black, acutely protruding outwards between M_1_ and M_3_; a pale yellow band distally of postmedial line, scattered with black scales; submarginal line and fringe similar to those of forewing; terminal line less distinct than that of forewing; discal spot smaller. Underside pale yellow, transverse lines dark grey, similar to those of upper side, discal spots black, more distinct than those of upper side.

Abdomen. Dorsum greyish white, dotted with black scales, anal tuft pale yellow. Setal patch absent on third sternite of male abdomen.

Male genitalia. Uncus with bifurcate apex, about two-thirds length of basal width. Gnathos with median process slender, pointed terminally, equal to length of uncus. Valva compressed, about twice as long as basal width; costa sclerotized, straight, bearing long setae from center to apex. Saccus semicircular. Juxta long and broad, terminal half triangular, with pointed apex. Coremata not developed. Aedeagus cylindrical, striated posteriorly; vesica scobinate, without cornuti.

#### Diagnosis.

This species is similar to *Biston suppressaria* (see below).

#### Material examineds.

**CHINA, Hainan** (IZCAS): Ledong, Jianfengling, Tianchi, 808 m, 18.V.2009, coll. Chen Fuqiang, 1♂.

#### Distribution.

China (Hainan); S. Thailand, Peninsular Malaysia, Sundaland.

#### Remarks.

The Hainan specimen is different from specimens of Bornean material in the distinct transverse lines and the more acute projection between M_1_ and M_3_ of the hindwing postmedial line. However, the male genitalia of the Hainan specimen are almost indentical to those of *Biston pustulata* from Borneo which were illustrated by Holloway (1994). Thus we classify the Hainan specimen as *Biston pustulata*. If these differences prove constant in a larger number of specimens, the Hainan population should be described as a separate subspecies.

**Figures 31–42.**
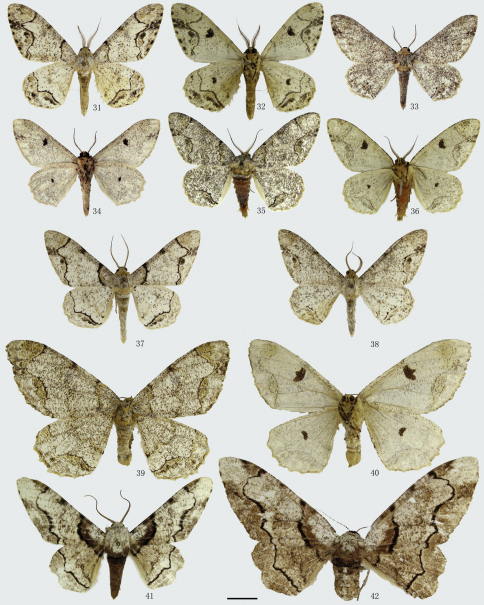
Adults of *Biston*. **31**–**32** *Biston pustulata*. **31** male **32** ditto, underside. **33**–**40** *Biston suppressaria*. **33** male (holotype of *Biston suppressaria benesparsa*) **34** ditto, underside **35** male (Cili, Hunan) **36** ditto, underside **37** male (Diaoluoshan, Hainan) **38** male (Bawangling, Hainan) **39** female **40** ditto, underside; **41**–**42** *Biston regalis*. **41** male **42** female. Scale bar = 1 cm.

### Biston suppressaria

(Guenée, 1858)

http://species-id.net/wiki/Biston_suppressaria

Figs 33–40 80 107 123

Amphidasys suppressaria Guenée, 1858, *in* Boisduval & Guenée, *Hist. nat. Insectes* (Spec. gén. Lépid.), 9: 210. Syntypes 1♂, 2♀, central India.
Buzura multipunctaria  Walker, 1863, *List Specimens lepid. Insects Colln Br. Mus.*, 26: 1531. Syntypes 3♂♀, Silhet? (BMNH)
Biston suppressaria : Hampson, 1895, *Fauna Br. India *(Moths), 3: 247.
Buzura suppressaria : Prout, 1915, *in* Seitz, *Macrolepid. World*, 4: 360, pl. 19: i.
Buzura suppressaria benescripta Prout, 1915, *in* Seitz, *Macrolepid. World*, 4: 360. Holotype ♂, China: Chung-king. (BMNH), syn. n.
Biston (Buzura) suppressaria : Wehrli, 1941, in *Seitz, Gross-Schmett. Erde*, 4 (Suppl.): 436.
Biston (Buzura) suppressaria f. benesparsa Wehrli, 1941, *in* Seitz, *Gross-Schmett. Erde*, 4 (Suppl.): 436, pl. 36: f. Holotype ♂, China: Hunan, Höng-shan. (ZFMK)
Biston luculentus Inoue, 1992, *Bull. Fac. domest. Sci. Otsuma Wom. Univ.*, 28: 171, figs 59, 60, 62–64. Holotype ♂, Thailand: Chanthaburi, Khao Soi Dao, 400 m. (UOS), syn. n.

#### Diagnosis.

This species is very close to *Biston pustulata*, but we can distinguish it by the following characters: the protrusion between M_1_ and M_3_ of the forewing postmedial line is shorter, shallowly bilobed and sometimes round; the projection between M_1_ and M_3_ of the hindwing postmedial line is round. In the male genitalia, it differs in the round apex of the juxta. The diagnostic characters of the female genitalia can be seen in *Biston bengaliaria*.

#### Material examineds.

**CHINA, Hunan **(ZFMK): Höng-shan, 900m, 25.V.1933, coll. H. Höne, 1♂ (Holotype of *Biston suppressaria benesparsa*). **Henan**: Lingshan, 350 m, 25.V.1999, coll. Shen Xiaocheng, 1♂. **Shaanxi** (IZCAS): Foping, 900 m, 27.VI.1999, coll. Zhu Chaodong, 1♂. **Jiangsu** (IZCAS): 2.VIII.1933, 1♂1♀. **Anhui** (IZCAS): Huainan, 19.VII–2.VIII.1981, 2♂. **Zhejiang** (IZCAS): Hangzhou, 19.VII–15.VIII.1972, coll. Liu Youqiao, 7♂; Hangzhou, 16.VII–1.VIII.1973, coll. Liu Youqiao and Zhang Baolin, 6♂1♀; Hangzhou, 22.V–17.VI.1976, coll. Chen Yin, 1♂; Tianmushan, V.1936, 2♀; Tianmushan, 30.VII.1972, coll. Liu Youqiao, 2♂; Tianmushan, 24–25.VII.1973, coll. Zhang Baolin, 2♂; Taishun, Siqianzhen, 250 m, 4.VIII.2005, coll. Lang Songyun, 12♂; Taishun, Wuyanling, 400–680 m, 29.VII.–1.VIII.2005, coll. Lang Songyun, 10♂; **Hubei** (IZCAS):Zigui, Jiulingtou, 220–300 m, 23.VI–25.VII.1993, coll. Song Shimei and Yao Jian, 8♂1♀; Xingshan, Longmenhe, 1350 m, 21.VI–25.VII.1993, coll. Song Shimei and Huang Runzhi, 3♂; Badong, Sanxia Linchang, 180 m, 13–14.V.1994, coll. Li Wenzhu, 2♂; Dangyang, 1980, 1♂. **Jiangxi** (IZCAS): Dayu, 18.VII.1975, coll. Song Shimei and Zhang Baolin, 2♂; Jiulianshan, 30–31.VII.1975, coll. Song Shimei, 4♂; Dadishan, X.1980, 1♂; Xinyu, VII.1980, 1♂; Boyang, VII.1980, 1♂. **Hunan** (IZCAS): Changsha, Weishengwusuo, 3.V.1979, coll. Gan Yuankai, 1♂; Yueyang, VI.1981, 1♂; Cili, IV–17.V.1981, 2♂; Fenghuang, 16.IX.1988, coll. Song Shimei, 1♀. **Fujian** (IZCAS): Mt. Wuyi, Huangxizhou, 500 m, 29.VII.2006, coll. Yang Chao, 1♂; Nanjing, 24.VII.1973, coll. Chen Yixin, 1♂; Sanming, 13.VII–29.VIII.1981, coll. Xiao Hu, 3♂; Jiangle, Longqishan, 10.VIII.1991, coll. Song Shimei, 1♂. **Guangdong** (IZCAS): Boluo, III.1973, 3♂; Guangzhou, 29.V.1973, coll. Zhang Baolin, 1♂; Guangzhou, VII.1985, coll. Su Xing, 1♂; Yingde, Chayesuo, 30.VII.1979, 1♂; Zhiwuyuan, 13.VI.1978, 1♂. **Hainan** (IZCAS): Danzhou, Liangyuan, 130 m, 16.V.2007, coll. Han Hongxiang and Lang Songyun, 3♂1♀; Limushan, 26.V.1984, 1♀; Qiongzhong, Limuling, 14.V.2007, coll. Han Hongxiang, 1♂; Baisha, Yinggeling, 434 m, 4–5.IV.2008, coll. Lang Songyun, 7♂1♀; Wuzhishan, Shuiman, 730–900 m, 3–8.V.2007, coll. Lang Songyun, 3♂; Tongshi, 340 m, 28.V.1973, coll. Chen Yixin, 1♂; Baoting, 80 m, 23.V.1973, coll. Chen Yixin, 1♂; Wanning, 60 m, 13.VI–29.VII.1963, coll. Zhang Baolin, 3♂1♀; Wanning, Xinglong, 41 m, 21.III.2008, coll. Lang Songyun, 1♂; Ledong, Jianfengling, 70–828 m, 17–21.V.2009, coll. Yan Keji, 11♂; Ledong, Jianfengling, 828 m, 24–27.III.2008, coll. Lang Songyun, 1♂1♀; Lingshui, Diaoluoshan, 260 m, 5.V.2007, coll. Han Hongxiang and Lang Songyun, 3♂1♀. **Guangxi** (IZCAS): Longsheng, 10–11.VI.1980, coll. Wang Linyao, 6♂1♀; Guilin, Yanshan, 19.VII.1976, coll. Zhang Baolin, 1♂; Guilin, 23.VI.1980, coll. Zhang Baolin, 1♂; Yulin, 400 m, 15.X.1983, coll. Luo Zhubiao, 1♂; Jinxiu, 200–1100 m, 10–20.V.1999, coll. Han Hongxiang et al., 14♂; Napo, Baihe, 440 m, 7.IV.1998, coll. Wu Chunsheng, 1♂; Qinzhou, 15.IV.1980, coll. Cai Rongquan, 1♂; Nanning, 13.IV.1980, 2♂; Pingxiang, 230 m, 8–17.VI.1976, 5♂2♀. **Sichuan** (IZCAS):Mt. Emei, Qingyinge, 800–1000 m, 20.V.1957, coll. Zhu Fuxing, 1♀; Yibin, Cuipingshan, 13.VIII.1981, coll. Zhang Yuelan, 1♂. **Guizhou** (IZCAS): Shibing, Ganxi, 690 m, 29.IV.1979, coll. Liu Wanzhao, 1♀; Jiangkou, Fanjingshan, 500 m, 11.VII.1988, coll. Li Wei, 3♂1♀. **Yunnan** (IZCAS): Baoshan, Bawan, 1100 m, 19–23.V.1992, coll. Xue Dayong, 2♂; Yongshan, 800 m, 30.IV.1979, 1♀; Lushui, 1250 m, 26.IV.1979, 1♀; Yuxi, Yuanjiang, 4.VII.1978, coll. Jiang Zhaolong, 1♂; Funing, Boyi, 250 m, 17.IV.1998, coll. Zhu Chaodong, 1♂; Cangyuan, 750 m, 19–22.V.1980, coll. Li Hongxing et al., 3♂; Ruili, Dengga, 980 m, 11.V–8.VI.1992, coll. Xue Dayong, 3♂; Wanding, 400 m, 10.VI.1992, coll. Xue Dayong, 1♂; Xinping, Mosha, 800 m, 9.VIII.1980, 2♂; Jingdong, 1170 m, 21.IV–9.VI.1956, coll. Zagwryyw, 2♂; Xishuangbanna, Damenglong, 650 m, 13.VIII.1958, coll. Zhang Yiran, 1♂; Xishuangbanna, Mengzhe, 1200 m, 8.IX.1958, coll. Wang Shuyong, 1♀; Xishuangbanna, 580–700 m, 13–14.IX.1993, coll. Xu Huanli et al, 3♂; Yiwu, Banna, Menglun, 650 m, 29.IV–2.V.1964, coll. Zhang Baolin, 2♀. **Tibet** (IZCAS): Mêdog, Yarang, 1091 m, 20–23.VIII.2006, coll. Lang Songyun, 1♂. More material from Zhejiang, Hubei, Guangdong, Sichuan and Yunnan in coll. ZFMK.

#### Distribution.

China (Henan, Shaanxi, Jiangsu, Anhui, Zhejiang, Hubei, Jiangxi, Hunan, Fujian, Guangdong, Hainan, Hong Kong, Guangxi, Sichuan, Chongqing, Guizhou, Yunnan, Tibet); India; Burma; Nepal.

#### Remarks.

Prout (1915) mentioned that *Biston suppressaria benescripta* (Prout, 1915) can be distinguished from *Biston suppressaria suppressaria* (Guenée, 1858) by the more obvious transverse lines, the absence of the median yellow band and the sparser black dots on the wings. However, after the examination of a long series of material, we find that the form with these variations occurs sympatrically with the nominotypical subspecies, such as in Hunan (Figs 33, 35) and Hainan (Figs 37, 38). And there is no genital difference between the two subspecies. So, we treat *Biston suppressaria benescripta* as a junior synonym of *Biston suppressaria suppressaria*.

*Biston luculentus* Inoue, 1992, described from SE. Thailand, is similar to *Biston suppressaria benescripta*, but has the transverse lines even more clearly expressed (e.g. see fig. 37 which is almost identical with *Biston luculentus*). Like *Biston suppressaria benescripta*, also the *Biston luculentus* form occurs sympatrically with typical *Biston suppressaria suppressaria* or with *Biston suppressaria benesparsa* Wehrli, the latter being a rather rare form, at many places. Also at the type locality of *Biston luculentus* (Prov. Chanthaburi, Khao Soi Dao) it occurs together with typical *suppressaria* (coll. ZFMK) Comparison of the genitalia of the two revealed no differences. Thus we follow Stüning (in litt.) and synonymize *Biston luculentus* with *Biston suppressaria*. Besides, we also believe that the strange, almost patternless female figured by Inoue (1992) as paratype of *Biston luculentus*, belongs to another, still unidentified species.

### Biston regalis

(Moore, 1888)

http://species-id.net/wiki/Biston_regalis

Figs 41, 42 81 108 124

Amphidasys regalis Moore, 1888, *in* Hewitson & Moore, *Descr. new Indian lepid. Insects Colln late Mr W.S. Atkinson*, (3): 234. Syntypes ♂♀, India: Darjeeling. (BMNH)
Biston regalis : Prout, 1915, *in* Seitz, *Macrolepid. World*, 4: 359, pl.19: h.

#### Diagnosis.

The wing pattern of this species is similar to that of *Biston exalbescens* Inoue, 2000 (Philippines) as follows: the forewing postmedial line is weakly waved, broadly protruding outwards between R_5_ and M_3_ and below CuA_2_; the hindwing outer margin is concave between M_1_ and M_3_; the hindwing postmedial line protrudes outwards between M_1_ and M_3_; dark brown bands are present basally of the forewing antemedial line and distally of the postmedial line of both wings, and usually absent at apical area and between M_3_ and CuA_1_ of the forewing. But the species can be distinguished from *Biston exalbescens* by the following characters: the forewing antemedial line is thinner, the dark brown band basally of it is narrower; the medial lines of both wings are less conspicuous. In the male genitalia, it differs in the much stronger central setose area of the valva; the median process of the gnathos is spatulate terminally, but pointed in *Biston exalbescens*; the juxta is longer and narrower; the vesica with two cornuti, a basal, oval plate with a lateral tooth and an elongate, sclerotized, spined fold. The female genitalia of this species are close to that of *Biston betularia*, but it has a nearly triangular lamella postvaginalis, which is absent in *Biston betularia*; the ductus bursae is broader and sclerotized, without antrum; the corpus bursae is pouched, but enlarged posteriorly and narrow medially in *Biston betularia*; the signum is almost oval, but bar-like in *Biston betularia*.

#### Material examineds.

**CHINA,** **Liaoning** (IZCAS): Fengcheng, Sitaizi, 23.VII.1982, coll. Song Shimei, 2♂. **Henan** (IZCAS):Nanyang, Baiyunshan, 1300 m, 1.VI.2001, coll. Shen Xiaocheng, 1♂; Nanyang, Baiyunshan, 1400 m, 21–27.VII.2003, coll. Zhang Dandan, 2♂; Nanyang, Baotianman, 24.VI.2006, coll. Shen Xiaocheng, 1♂; Nanyang, Baotianman, 623 m, 12.VIII.2008, coll. Xue Dayong, 1♂; Yanshi, 21.VI.1981, 1♂. **Shaanxi** (IZCAS):Foping, 950 m, 24–25.VII.1998, coll. Yuan Decheng, 4♂; Ningshaan, Huoditang, 1580 m, 19.VIII.1998, coll. Yuan Decheng and Zhang Xuezhong, 3♂;Liuba, Miaotaizi, 1020–1350 m, 18–21.VII.1998, coll. Yao Jian and He Tongli, 5♂; Zhouzhi, Houzhenzi, 1350 m, 24.VI.1999, coll. Zhu Chaodong, 1♂; Huangbaiyuan, 1000 m, 13–18.VII.1980, coll. Han Yinheng and Zhang Baolin, 4♂. **Gansu** (IZCAS): Yongdeng, Tulugou, 2280 m, 4.VI.1992, coll. Xue Dayong, 1♂; Tianshui, Longmen Linchang, 1990, 1♂; Chengxian, Feilongxia, 1020 m, 4.VII.1999, coll. Yao Jian, 2♂; Kangxian, Baiyunshan, 1250–1750 m, 12.VII.1998, coll. Wang Shuyong and Yao Jian, 2♂; Kangxian, Qinghe Linchang, 1450–1650 m, 4–8.VII.1999, coll. Yao Jian and Zhu Chaodong, 3♂; Kangxian, Yangba, 1000 m, 10–11.VII.1999, coll. He Tongli and Zhu Chaodong, 2♂; Wenxian, Tielou, 1450 m, 24.VII.1999, coll. Zhu Chaodong, 3♂; Wenxian, Qiujiaba, 2350 m, 22.VII.1999, coll. Yao Jian, 2♂. **Zhejiang** (IZCAS): Tianmushan, 21.VII.1973, coll. Zhang Baolin, 1♂. **Hubei** (IZCAS): Shennongjia, Songbai, 950 m, 14–18.VII.1980. coll. Yu Peiyu, 3♂; Shennongjia, Dajiuhu, 1800 m, 1.VIII.1981, coll. Han Yinheng, 1♂; Shennongjia, Jiuchong, 700 m, 1.VII.1998, coll. Ye Chanjuan, 1♂; Hefeng, Fenshuiling, 1240 m, 29–31.VII.1989, coll. Li Wei and Yang Longlong, 9♂1♀; Xingshan, Longmenhe, 1300–1350 m, 21.VI–18.VII.1993, coll. Song Shimei and Huang Runzhi, 5♂; Lichuan, Xingdoushan, 800 m, 21–23.VII.1989, coll. Li Wei and Yang Longlong, 2♂. **Jiangxi** (IZCAS): Guling, 13.VII.1935, 1♂; Lushan, 5.VII.1975, coll. Liu Youqiao, 1♂. **Hunan** (IZCAS): Sangzhi, 8.VII.1981, 2♂. **Fujian** (IZCAS): Mt. Wuyi, 21.IX.1982, coll. Zhang Baolin, 2♂. **Hainan** (IZCAS):Qiongzhong, Limushan, 647 m, 29.XII.2007, coll. Li Jing, 1♂; Bawangling, Donger Linchang, 1015 m, 8–10.V.2007, coll. Chen Fuqiang, 1♂; Baisha, Yinggeling, Yinggezui, 619 m, 17–19.XI.2009, coll. Yang Chao, 1♂; Wuzhishan, Shuiman, 730–900 m, 8.V.2007, coll. Lang Songyun, 1♂; Jianfengling, 18.V.1982, coll. Chen Zhiqing, 1♀; Ledong, Jianfengling, 934 m, 14–17.XII.2007, coll. Li Jing, 4♂; Ledong, Jianfengling, 934 m, 14–17.XII.2007, coll. Li Jing, 1♂; Lingshui, Diaoluoshan, 920 m, 3.V.2007, coll. Lang Songyun, 1♂; Lingshui, Diaoluoshan, 929 m, 11–12.XII.2007, coll. Li Jing, 2♂. **Sichuan** (IZCAS): Mt. Emei, Qingyinge, 800–1000 m, 29.VI–15.VII.1957, coll. Yu Youcai, 4♂; Xichang, Lushan, 8.VIII.1980, 1♂; Dukou, Pingdi, 28.VI.1981, coll. Zhang Baolin, 2♂. **Yunnan** (IZCAS): Weixi, 2320 m, 24.VI.1979, coll. Yan Xiangqun, 1♂; Baoshan, Bawan, 1100 m, 19–23.V.1992, coll. Xue Dayong, 1♂; Tengchong, Heinitang, 1930 m, 28–30.V.1992, coll. Xue Dayong, 5♂; Xiaomenglun, 7.V.1980, 1♂; Qujing, 7.IX.1982, coll. Fang Chenglai, 1♂. More material from Shaanxi, Zhejiang, Hubei and Yunnan, in coll. ZFMK.

#### Distribution.

China (Liaoning, Henan, Shaanxi, Gansu, Zhejiang, Hubei, Jiangxi, Hunan, Fujian, Taiwan, Hainan, Sichuan, Yunnan), Russia (Amur, Ussuri), Japan, North Korea, South Korea, India, Nepal, Philippines, Pakistan, United States.

#### Remarks.

The subspecies of *Biston regalis* from China is *Biston regalis comitata *(Warren, 1899) (*Eubyjodonta comitata *Warren, 1899, *Novit. zool.*, 6: 50. Syntypes 2♂, Russia: Amurland, Sidemi. (BMNH)).

### Biston brevipennata

Inoue, 1982

http://species-id.net/wiki/Biston_brevipennata

Figs 43 82 109

Biston brevipennata Inoue, 1982b, *Bull. Fac. domestic Sci., Otsuma Woman’s Univ*., 18: 176, figs 40e, 41b. Holotype ♂, Nepal: Lete, 2400 m near Nilgiri. (BMNH)

#### Diagnosis.

The present species can be distinguished from the other species in group II by the following characters: smaller sized (length of forewing: 23–25 mm in male); the forewing outer margin is less waved; the band basally of the forewing antemedial line is much thinner; the speckles scattered on the wings are dark brown, not black. In the male genitalia, the apex of the uncus is broader and shallowly bifurcated but narrower and round in the others; the juxta is more sharply pointed at tip.

#### Material examineds.

**CHINA, Tibet **(IZCAS): Gyirong, 18.VI.–23.VII.1984, coll. Yan Zhaoxing and Pu Qiongzhi, 2♂; Zham, 2200 m, 25.VI.1975, coll. Wang Ziqing, 1♂.

#### Distribution.

China (Tibet), Nepal.

**Figures 43–55.**
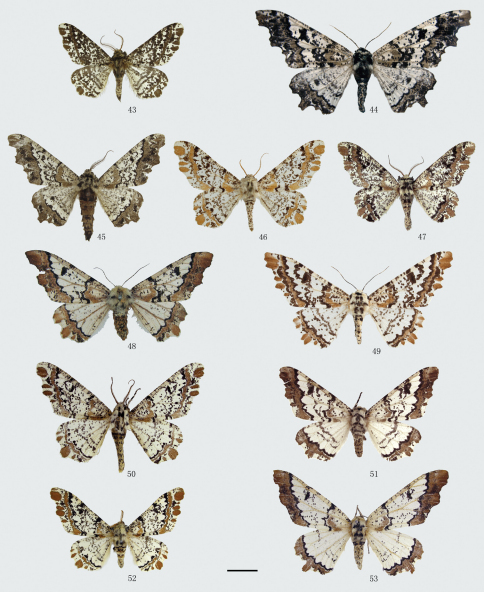
Adults of *Biston*. **43** *Biston brevipennata*, male **44**–**45** *Biston quercii*. **44** female (holotype) **45** male **46**–**53** *Biston falcata*. **46** male (holotype of *Amphidasis erilda*) **47** male (holotype of *Biston erilda satura*) **48** female (holotype of *Biston emarginaria*) **49 **female (holotype of *Amphidasis clorinda*) **50** male (Lijiang, Yunnan) **5**1 female (Gyirong, Tibet) **52 **male (Zhouqu, Gansu) **53** female (Zhouqu, Gansu). Scale bar = 1 cm.

### Biston quercii

(Oberthür, 1910)

http://species-id.net/wiki/Biston_quercii

Figs 44, 45 83 110

Amphidasis quercii Oberthür, 1910, *Études Lépid. comp*., 4: 676, pl. 51, fig. 433. Holotype ♀, China: Sichuan, Tien-Tsuen. (ZFMK)
Biston quercii : Prout, 1915, *in* Seitz, *Macrolepid. World*, 4: 359.
Biston (Eubyjodonta)quercii : Wehrli, 1941, *in* Seitz, *Gross-Schmett. Erde*, 4 (Suppl.): 434, pl. 36: f.

#### Diagnosis.

The wing pattern of this species is similar to that of *Biston falcata* as follows: the forewing antemedial line is black, slightly waved; the postmedial lines of both wings are black and dentate; broad brown bands are present basally of the forewing antemedial line and distally of the postmedial lines of both wings; the speckles scattered on the wings are black, and often gather to a black patch basally of the submarginal lines; the hindwing medial line is black and double. But it can be distinguished by the following characters: the outer margins are more undulating, there are distinct marginal processes in the centre of both wings, absent in *Biston falcata*; the hindwing discal spot is present. The male genitalia are similar to those of *Biston falcata* as follows: the apex of the uncus is round; the median process of the gnathos is broad and round apically; the juxta is long, narrow, acute and with a longitudinal arris apico-ventrally; the cornutus is shaped as a spinous patch. But this species is characterized by the narrower juxta and the longer spines of the cornutus.

#### Material examineds.

**CHINA, Sichuan **(ZFMK): Tien-Tsuen, 1901, coll. native collectors (chasseurs indigènes de feu le P. Déjean), 1♀ (Holotype). **Henan**(IZCAS): Baiyunshan, 1300 m, 19.VII.2002, 18.VI.2003, coll. Shen Xiaocheng, 2♂. **Shaanxi**(IZCAS): Ningshaan, Yaquegou, 1580–1750 m, 7.VII.1999, coll. Yuan Decheng, 1♂. **Gansu**(IZCAS): Kangxian, Qinghe Linchang, 1450–1650 m, 15.VII.1998, coll. Yao Jian and Wang Hongjian, 4♂. **Hubei**(IZCAS): Shennongjia, Dajiuhu, 1800m, 1.VIII.1981, coll. Han Yinheng, 1♂; Xingshan, Longmenhe, 1350 m, 14–16.VII.1993, coll. Song Shimei, 4♂. **Sichuan**(IZCAS): Wenchuan, Wolong, 1920 m, 22.VII.1983, coll. Wang Shuyong, 1♂. A few males from Shaanxi and Sichuan in coll. ZFMK.

#### Distribution.

China (Henan, Shaanxi, Gansu, Hubei, Sichuan).

### Biston falcata

(Warren, 1893)

http://species-id.net/wiki/Biston_falcata

Figs 46–53 84–88 111–113 125–127

Eubyjodonta falcata Warren, 1893, *Proc. zool. Soc. Lond*., 1893 (2): 416. Syntypes ♂, India: Sikkim. (BMNH)
Biston falcata : Hampson, 1895, *Fauna Br. India *(Moths), 3: 246.
Biston emarginaria Leech, 1897, *Ann. Mag. nat. Hist.*, (6) 19: 322, pl. 7, fig. 8. Holotype ♀, China: Sichuan, Pu-tsu-fong. (BMNH), syn. n.
Amphidasis erilda Oberthür, 1910, *Études Lépid. comp*., 4: 677, pl. 51, fig. 435. Holotype ♂, China: Yunnan, Tse-Kou. (ZFMK) , syn. n.
Amphidasis clorinda Oberthür, 1910, *Études Lépid. comp.*, 4: 677, pl. 51, fig. 434. Syntype(s), China (west): Tse-Kou, syn. n.
Biston falcata clorinda : Parsons et al., 1999, *Geometrid Moths of the World, a Catalogue*, 1: 86.
Biston falcata satura Wehrli, comb. n.

#### Diagnosis.

The diagnostic characters of the external mophology and the male genitalia can be seen in the preceding species. In the female genitalia, the species can be distinguished from other congeners by the following characters: the apophyses posteriores are short, about twice the length of apophyses anteriores; the ductus bursae is sclerotized and striated longitudinally, about one-third the length of corpus bursae; the corpus bursae is weakly sclerotized and striated posteriorly; the signum usually consists of two small separate sclerotized patches.

#### Material examineds.

**CHINA, Yunnan** (ZFMK): Tse-kou, coll. R. P. Dubernard, 1895, 1♀ (Holotype of *Biston falcata clorinda*); Tse-kou, coll. R. P. Dubernard, 1900, 1♂(Holotype of *Amphidasis erilda*); **Shaanxi **(ZFMK): Tapai-shan im Tsinling, Sued-Shensi, 25.VI.1935, coll. H. Höne, 1♂ (Holotype of *Biston erilda satura*); **Zhejiang **(ZFMK):West-tien-mu-shan, 1♂; **Sichuan** (ZFMK): Chasseurs Indigènes de Ta-tsien-lou, Récolte de 1910,1♀; **Sichuan** (BMNH): Pu-tsu-fong,2993 m, VI–VII.1890, coll. Native, 1♀. (Holotype of *Biston emarginaria*). **Ningxia**(IZCAS): Jingyuan, 2052–2084 m, 29.VI.–2.VII.2008, coll. Sun Wenhui, 3♂; Longde, Sutai Linchang, 2165 m, 6.VII.2008, coll. Song Wenhui, 2♂. **Gansu**(IZCAS): Zhouqu, Shatan Linchang, 4–7.VII.1998, coll. Yao Jian et al., 17♂;Zhouqu, Shatan Linchang, 2400 m, 4–16.VII.1999, coll. Yao Jian et al., 17♂4♀;Wenxian, Baishuijiang, IX.1986, coll. Wang Hongjian, 1♂; Wenxian, Qiujiaba, 2350 m, 21–29.VII.1999, coll. Chen Jun et al., 9♂; Shalin, 2357 m, 25.VI–28.VII.2002, coll. Cao Xiuwen, 2♂; Wushan, Tanyu, 26.VII.1990, coll. Deng Lianhai, 1♂; Kangxian, Qinghe Linchang, 1400 m, 8.VII.1999, coll. Zhu Chaodong, 1♀; Renmingchi, 2450 m, 30.VII.2002, 1♀; Baishuijiang, VII.1986, coll. Wang Hongjian, 1♀. **Sichuan**(IZCAS):Wenchuan, Wolong, 1920 m, 24–29.VII.1983, coll. Wang Shuyong et al., 4♂; Wenchuan, Wolong, 1900 m, 27.VIII.1982, coll. Chen Yuanqing, 1♀; Mt. Emei, Jiulaodong, 1800–1900 m, coll. Huang Keren, 2♂; Gonggashan, Yanzigou, 2350 m, 3.VI.1983, coll. Zhang Xuezhong, 1♂; Luding, Moxi, Hailuogou, 2821–3155 m, 15–17.V.2009, coll. Li Jing, 2♂. **Yunnan**(IZCAS): Lijiang, Yulongshan, 2700–2800 m, 27.VII.1984, coll. Chen Yixin and Liu Dayun, 3♂; Lijiang, Yulongshan, 2680–3296 m, 15–23.VI.2009, coll. Han Hongxiang et al., 18♂; Jizushan, 2500 m, 30.VI.1980, 1♂; Yiliang, 200 m, 13.VII.1979, 1♂; Yiliang, 20.VII.1982, coll. Wang Linyao, 1♂; Diqing, Xiaozhongdian, Linyechang, 3700 m, 28.VII.1979, coll. Yan Baiqun, 1♀; Xiaozhongdian, 3100 m, 30.VII.1984, coll. Liu Dayun, 1♂; Tengchong, 2500 m, 2–4.VI.1992, coll. Xue Dayong, 3♂. **Tibet** (IZCAS): Nyingchi, Pêlung, Mamba, 2115 m, 1–2.IX.2005, coll. Wang Xuejian, 1♂; Nyingchi, Bayi, 2999 m, 1–3.VIII.2006, coll. Lang Songyun, 2♂; Mainling, Pai, 2883 m, 4–6.VIII.2006, coll. Lang Songyun, 7♂; Bomi, Yi’ong, 2300–2750 m, 28–31.VIII.1983, coll. Han Yinheng and Lin Zai, 2♂; Bomi, Yi’ong, Tangmai, 2079 m, 29–30.VIII.2006, coll. Lang Songyun, 1♂; Mêdog, 1060–3213 m, 7–13.VIII.2006, coll. Lang Songyun, 3♂; Cona, Mama, 2900 m, 6.VIII.1974, coll. Huang Fusheng, 1♀; Nyalam, Zham, 2250 m, 9–20.V.1974, coll. Zhang Xuezhong, 3♂; Zham, Qu, 3300 m, 6–7.VII.1975, coll. Huang Fusheng and Wang Ziqing, 2♂; Gyirong, 2800–3300 m, 26.VII–8.VIII.1975, coll. Wang Ziqing and Huang Fusheng, 3♂3♀; Gyirong, Gongshe, 28.VIII.1984, coll. Yan Zhaoxing, 1♂; Yadong, 2800 m, 23.VII.1960, coll. Wang Chunguang, 1♂; Yadong, 10.VIII.1982, coll. Wangjia Tsering, 2♂; Yadong, Linchang, 29.VIII.1984, Li Aihua, 1♀. Large series of material from Yunnan (ssp. *falcata*) and Shaanxi (ssp. *satura* Wehrli) in coll. ZFMK.

#### Distribution.

China (Shaanxi, Ningxia, Gansu, Zhejiang, Sichuan, Yunnan, Tibet), India, Nepal.

#### Remarks.

After examinating the types of *Amphidasis erilda* Oberthür, 1910, *Amphidasis clorinda* Oberthür, 1910, *Biston emarginaria* Leech, 1897, *Biston erilda satura* Wehrli, 1941 and a large series of material from China and the neighbouring regions it became obvious that *Biston emarginaria *(only females known) and *Amphidasis clorinda* (female holotype known only) are all females of *Amphidasis erilda*. The external and genital features of *Amphidasis erilda*, on the other hand, turned out to be almost identical or fall within the range of variation of *Biston falcata*. Thus we treat *Biston emarginaria*, *Amphidasis erilda*, *Amphidasis clorinda* as junior synonyms of *Biston falcata*. *Biston erilda satura*, as described by Wehrli (1941), is treated as a valid subspecies, but has be combined newly with *Biston falcata*, as explained above. Thus, two Chinese subspecies of *Biston falcata* are *Biston falcata falcata* (Warren, 1893) and *Biston falcata satura* (Wehrli, 1941). In China, the former is distributed in Sichuan, Yunnan and Tibet (Figs 46, 48–51, 84–86, 112, 125,126), the latter is distributed in Shaanxi, Ningxia and Gansu (Figs 47, 52, 53, 87, 88, 113, 127). There are some intraspecific variations between individuals of *Biston falcata*, for example, in the the male genitalia, the apical part of the valva varies from broad to narrow in the same region, such as Gansu (Figs 87, 88) and Tibet (Figs 85, 86); in the female genitalia, the signum usually consists of two small separate sclerotized patches, sometimes there is only one signum or the signum is very tiny, and the position of the signum is variable individually.

### Biston perclara

(Warren, 1899)

http://species-id.net/wiki/Biston_perclara

Fig. 54

Blepharoctenia perclara Warren, 1899, *Novit. zool*., 6: 49. Holotype ♂, China: Taiwan, Keelung. (BMNH)
Cusiala bengaliaria cerea Bastelberger, 1909, *Dt. ent. Z. Iris*, 22: 177. Holotype ♀, China: Taiwan. (Synonymized by Prout (1914))
Epamraica bilineata Matsumura, 1910, *Thousand Insects Japan*, (Suppl.) 2: 130, pl. 28, fig. 1. Syntypes ♀, Japan; China: Taiwan. (Synonymized by Prout (1914))
Biston perclara : Prout, 1914, *Ent. Mitt*., Berlin 3: 264.

#### Diagnosis.

The external characters of this species are similar to those of *Biston thibetaria* as follows: the forewing antemedial line and the postmedial lines of both wings are black and thick; black patches are present distally of the forewing postmedial line between M_1_ and M_3_, reaching the outer margin; another smaller black patch is present distally of the forewing postmedial line below M_3_. But in this species the broad bands placed basally of the antemedial line of the forewing and distally of the postmedial lines of both wings are pale yellow and indistinct, whereas they are yellowish green and distinct in *Biston thibetaria*; the discal spots of both wings are less distinct or have completely vanished; the forewing postmedial line is straight between CuA_2_ and 1A + 2A, while it is slightly excurved in *Biston thibetaria*. The male genitalia of the species are almost identical to those of *Biston thibetaria.* But the median process of the gnathos of the species is truncate apically and the incision of posterior margin of the juxta is less deep.

#### Material examineds.

**CHINA, Taiwan**(ZFMK): Hueison Forest, Nantou, 570–800 m, 28–29.IX.1992, coll. F. Aulombard and J. Plante, 1♂. A large series from different localities of Taiwan in coll. ZFMK.

#### Distribution.

China (Taiwan), Japan.

### Biston thibetaria

(Oberthür, 1886)

http://species-id.net/wiki/Biston_thibetaria

Figs 55- 57 89 114 128

Amphidasys thibetaria Oberthür, 1886, *Études ent*., 11: 32, pl. 5, fig. 30. Holotype ♀, China: Sichuan (?), Châpa. (ZFMK)
Buzura thibetaria : Prout, 1915, *in *Seitz, *Macrolepid. World*, 4: 360, pl. 19: h.
Buzura (Blepharoctenia) thibetaria : Wehrli, 1941, *in* Seitz, *Gross-Schmett. Erde*, 4 (Suppl.): 436.
Biston thibetaria : Parsons et al., 1999, *Geometrid Moths of the World, a Catalogue*, 1: 88.

#### Diagnosis.

This species is very distinct and is easily recognizable by the thick black lines and yellowish green bands placed basally of the antemedial line of the forewing and distally of the postmedial lines of both wings, the large, black ringed and pale-centred discal spots on both wings, as well as the black-belted abdomen and the fresh yellow anal tuft. The male genitalia of *Biston thibetaria* are close to those of *Biston panterinaria*: the apex of the uncus is bifurcated and about four-fifths as long as the basal width; the median process of the gnathos is short and round apically; the valva is broad basally and narrow apically; the ventral margin of the valva is slightly sinuous; the juxta has a deep incision at the middle on the posterior margin; the cornutus is stick-like; a narrow sclerotized band is present on lateral side of the aedeagus. But it can be distinguished from that species by the strongly rounded basal half of the valva. The female genitalia of the species are close to those of *Biston panterinaria* as follows: the ostium bursae is weakly sclerotized; the ductus bursae is very short; the corpus bursae is curved medially, striated in the posterior half and enlarged at tip; the signum is oval and with marginal spines. It differs in having an oval lamella postvaginalis, which is absent in *Biston panterinaria*.

#### Material examineds.

**CHINA, Sichuan **(ZFMK): Sichuan (?), Châpa, 1♂(Syntype). **Hubei** (IZCAS): Shennongjia, 600–700 m, 17–18.VII., 2.VIII.1998, coll. Ye Chanjuan, 3♂; Xingshan, Longmenhe, 730–1350 m, VI–VII.1993, coll. Song Shimei et al., 11♂1♀; Zigui, Jiulingtou, 220–250 m, 25.VII.1993, coll. Song Shimei, 2♂; Badong, 19.V.1989, 1♂; Hefeng, 650 m, 29.V.1989, coll. Li Wei, 1♂; Lichuan, Xingdoushan, 860 m, 6.VII.1989, coll. Li Wei, 1♂; Xianfeng, 800 m, 2.VI.1989, coll. Li Wei, 1♀. **Hunan** (IZCAS): Tianpingshan, 12.VIII.1981, 1♂. **Fujian** (IZCAS): Mt. Wuyi, Sangang, 7.VII.1982, coll. Wang Linyao, 1♂. **Guangxi** (IZCAS): Longsheng, 10–13.VI.1980, coll. Song Shimei and Wang Linyao, 4♂. **Sichuan** (IZCAS): Barkam, 2600 m, 21.VIII.1983, coll. Chai Huaicheng, 1♂; Luding, Moxi, 600–1900 m, 11–17.VI.1983, coll. Chai Huaicheng and Wang Shuyong, 3♂; Luding, Guzanjiangju, 1635 m, 21.V.2009, coll. Liang Hongbin and Wang Zhiliang, 1♂2♀; Batang, 1975, 9♂; Dukou, Pingdi, 5–22.VI.1987, coll. Zhang Baolin, 13♂; Huili, 23–29.VII.1974, coll. Han Yinheng, 2♂1♀; Yanyuan, Jinhe, 1230 m, 28.VI.1984, coll. Chen Yixin, 1♂. **Guizhou** (IZCAS): Meiyun, 6.IV.1978, coll. Xia Huai’en, 1♀. **Yunnan** (IZCAS): Lijiang, 22–23.V.1980, 7♂1♀; Lijiang, Yushuizhai, 2680 m, 21.VI.2009, coll. Qi Feng, 1♂; Qujing, 3–20.VII.1982, coll. Wang Linyao and Song Shimei, 4♂; Luoci, 21.VI.1982, coll. Song Shimei, 1♂; Yongsheng, Liude, 2250m, 9.VII.1984, coll. Liu Dayun, 1♂. More material from Hubei, Sichuan, Yunnan, Tibet in coll. ZFMK.

#### Distribution.

China (Henan, Zhejiang, Hubei, Hunan, Fujian, Guangxi, Sichuan, Guizhou, Yunnan, Tibet).

#### Remarks.

The female which Oberthür (1886, pl. 5, fig.30) figured, is generally considered to be the holotype of *thibetaria*. However, he indirectly mentioned a larger number of specimens in his original description, by writing: “some specimens have the wings crossed by curved medial lines which cut through the discoidal spots” (translated from French). The female is part of the ZFMK collection, as already mentioned by Wehrli (1941), but all other syntypes are not. It is well possible that they have been transferred to The Natural History Museum, London, which keeps a large part of the Charles Oberthür collection. If these specimens will be found existing, the female will lose its holotype status. Eight specimens of *thibetaria* from several Sichuan localities in the ZFMK collection bearing the typical printed labels of Oberthür have been collected later, in the years following the description of *thibetaria*, thus they do not belong to the syntype series.

**Figures 54–63.**
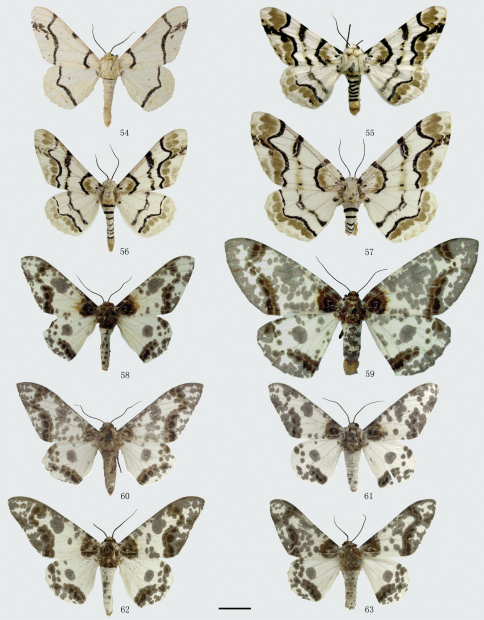
Adults of *Biston*. **54** *Biston perclara*, male **55**–**57** *Biston thibetaria*. **55** male (syntype) **56** male **57** female **58**–**63** *Biston panterinaria panterinaria*. **58** male (syntype of *Culcula* *panterinaria lienpingensis*) **59** female (syntype of *Culcula panterinaria szechuanensis*) **60** male (Dayu, Jiangxi) **61** male (Jiulianshan, Jiangxi) **62** male (Shixing, Guangdong) **63** male (Pengshui, Sichuan). Scale bar = 1 cm.

**Figures 64–69.**
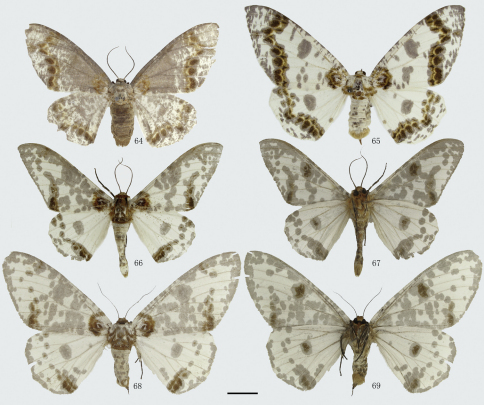
Adults of *Biston*. **64**–**65** *Biston panterinaria panterinaria*. **64** female (Fangshan, Beijing) **65** female (Yingtaogou, Beijing); **66**–**69** *Biston panterinaria exanthemata*. **66** male **67** ditto, underside **68** female **69** ditto, underside. Scale bar = 1 cm.

**Figures 70–75.**
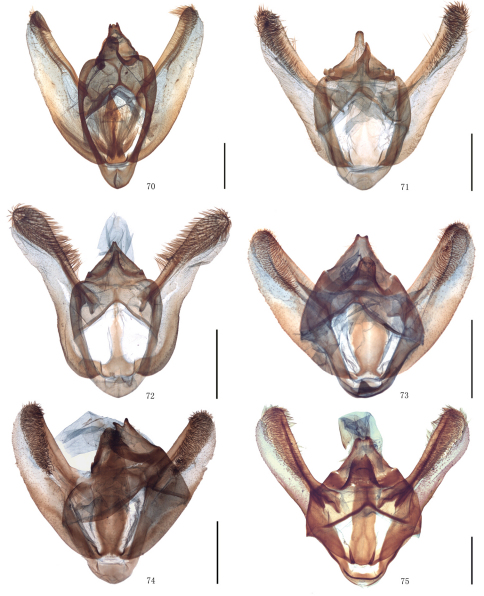
Male genitalia of *Biston*. **70** *Biston melacron* **71** *Biston marginata* **72** *Biston thoracicaria* **73** *Biston betularia parva* **74** *Biston betularia nepalensis* **75** *Biston robustum*. Scale bar = 1 mm.

**Figures 76–81.**
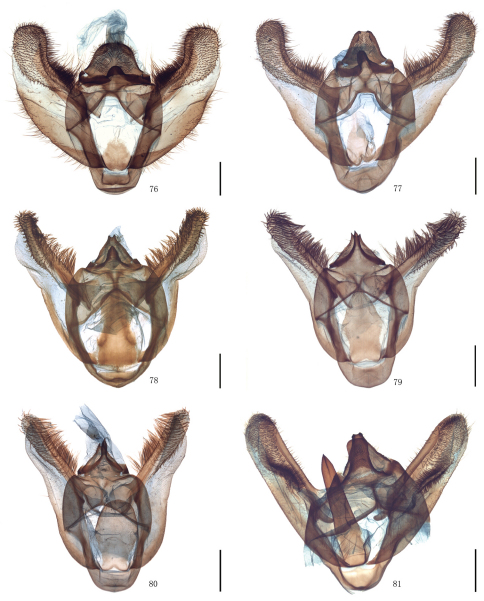
Male genitalia of *Biston*. **76** *Biston mediolata*sp. n. **77** *Biston contectaria* **78** *Biston bengaliaria* **79** *Biston pustulata* **80** *Biston suppressaria* **81** *Biston regalis*. Scale bar = 1 mm.

### Biston panterinaria

(Bremer & Grey, 1853)

http://species-id.net/wiki/Biston_panterinaria

Figs 58- 69 90- 96 115, 116 129, 130

Amphidasis panterinaria Bremer & Grey, 1853, *Beitr. Schmett.-Fauna nord. China*: 21, pl. 10, fig. 1. Syntypes, China (north).
Buzura abraxata Leech, 1889, *Trans. ent. Soc. Lond*., 1889 (1): 143, pl. 9, fig. 14. Syntype(s) ♀, China: Yangzee River, Kiukiang. (BMNH), syn. n.
Culcula panterinaria lienpingensis Wehrli, 1939, *in* Seitz, *Gross-Schmett. Erde*, 4 (Suppl.): 266, pl. 20: b. Syntype(s), China: Guangdong, Lienping. (ZFMK), syn. n.
Culcula panterinaria szechuanensis Wehrli, 1939, *in* Seitz, *Gross-Schmett. Erde*, 4 (Suppl.): 266, pl. 20: b. Syntype(s), China: Sichuan, Tien-Tsuen. (ZFMK), syn. n.
Culcula panterinaria : Inoue, 1946, *Bull. Lepid. Soc. Japan*,1 (2): 37.
Biston panterinaria : Sato, 1996, *Trans. lepid. Soc. Japan*, 47 (4): 223–236.

#### Diagnosis.

This species is different from other congeners by mimicking the pattern of distasteful or poisonous species of the genus *Abraxas* Leach. The wings are white and scattered with pale grey markings, which are rarely present basally of the hindwing postmedial line; the base of the forewing is grey and has a large brown patch, accompanied by the yellowish brown antemedial line; the postmedial lines of both wings are yellow, narrow and protruding outwards between M_1_ and M_3_, and diffused with dark brown oval patches; the discal spots of both wings are large pale grey dots; the discal spots on the underside of the wings are dark brown centrally. The diagnostic characters of genitalia can be seen in the previous species.

#### Material examineds.

**CHINA, Kwangtung [Guangdong]** (ZFMK): Lienping, 1♂ (Syntype of *Culcula* *panterinaria lienpingensis*). **Sichuan **(ZFMK): Tien-Tsuen, 1901, coll. native collectors (chasseurs indigènes de feu le P. Déjean), 1♂ (Syntype of *Culcula panterinaria szechuanensis*). **Liaoning** (IZCAS): Lingyuan, VI.1984, coll. Jin Laiwu, 1♂; Beipiao, VI.1984, coll. Liu Jin, 1♂; Dongling, 2♂. **Beijing** (IZCAS): Xiangshan, 9.VIII.1936, 1♂; Sanpu, 23.VIII.1972, coll. Zhang Baolin, 2♂; Fangshan, 26–29.VI.1972, coll. Zhang Baolin, 8♂1♀; Baihuashan, 30.VI.1972, 1♀; Pinggu, 9.VII.1975, 1♂; Jinshan, 17.VII.1988, 1♂; Jinshan, 6.VII.1990, 3♂; Huairou, 10.VII.1981, 1♂; Fengtai, 6.VII.1982, 1♂; Yingtaogou, 29.VI.1990, 1♀. **Hebei** (IZCAS): Zunhua, Dongling, 1♂. **Shanxi** (IZCAS): Taiyuan, 1♂. **Shandong** (IZCAS): Qufu, Shimensi, 17.VII.1980, 1♂. **Henan** (IZCAS): Linzhou, Shibanyan, 22.VII.2006, coll. Sun Hao, 1♂; Dengfeng, 8.VII.1981, 1♀; Baiyunshan, 1300 m, 13.VI.2001, coll. Shen Xiaocheng, 1♂; Baiyunshan, 1400 m, 26–28.VII.2003, coll. Zhang Dandan, 3♂; Baiyunshan, 1550m, 13–15.VIII.2008, coll. Song Wenhui, 1♂; Nanyang, Baotianman, 21.VI.2006, coll. Shen Xiaocheng, 1♂; Xinyang, Jigongshan, 250 m, 20–21.VII.2002, coll. Han Hongxiang, 1♂. **Shaanxi** (IZCAS): Dali, 10.VII.1981, 1♂; Ankang, 22.V.1981, 1♂; Changqing, Xihan, 9.V.1981, 1♂; Liuba, Miaotaizi, 1350m, 19–21.VII.1998, coll. Yao Jian and Zhu Chaodong, 20♂5♀; Foping, 890–900 m, 26–27.VI.1999, coll. Yao Jian, 1♂1♀. **Ningxia** (IZCAS):Jingyuan, Hongxia Linchang, 1998 m, 9.VII.2008, coll. Song Wenhui, 1♂. **Gansu** (IZCAS): Zhouqu, Shatan Linchang, 2350 m, 5.VII.1998, coll. Yuan Decheng, 1♂; Kangxian, Baiyunshan, 1250–1750 m, 12.VII.1998, coll. Wang Shuyong, 1♂; Kangxian, 1000–1400 m, 7–14.VII.1999, coll. Zhang Xuezhong et al., 10♂1♀. **Anhui** (IZCAS): Jiuhuashan, VII.1981, 1♂. **Zhejiang** (IZCAS): Moganshan, 11–12.V.1936, coll. O. Piel, 1♂1♀; Lin’an, VI.1981, 1♂; Tianmushan, 20–31.VII.1972, coll. Liu Youqiao et al., 11♂3♀; Lin’an, West Tianmushan, 400 m, 26–30.VII.2003, coll. Han Hongxiang and Xue Dayong, 18♂1♀; Hangzhou, 18.VII.1973, coll. Chen Ruijin, 1♂; Qingyuan, Fengyangshan, Datianping, 1290 m, 6–10.VIII.2003, coll. Han Hongxiang, 5♂; Taishun, Siqianzhen, 250–930 m, 31.VII.–4.VIII.2005, coll. Lang Songyun, 14♂. **Hubei** (IZCAS): Jingshan, 22.VII.1981, 1♂; Shennongjia, 950 m, 3–16.VII.1980, coll. Yu Peiyu, 8♂1♀; Shennongjia, 600–700 m, 17–20.VII.–7.VIII.1998, coll. Zhou Hongzhang et al., 11♂; Xingshan, Longmenhe, 1310 m, 17.VI.1993, coll. Li Wenzhu, 2♂; Xingshan, Xiaohekou, 700 m, 11.V.1994, coll. Li Wenzhu, 1♂; Lichuan, Xingdoushan, 800–860 m, 9.VI–23.VII.1989, coll. Li Wei and Yang Longlong, 19♂; Xianfeng, 800 m, 4.VI.1989, coll. Li Wei, 2♂; Hefeng, 1240 m, 28.VII.1989, coll. Li Wei, 1♂. **Jiangxi** (IZCAS): Fengxin, VIII.1980, 1♂; Yifeng, VIII.1979, 1♂; Jinggangshan, 2.VII.1975, coll. Zhang Baolin, 1♂; Jiulianshan, 27–31.VII.1975, coll. Song Shimei, 7♂; Doushui, 7.VII.1975, coll. Song Shimei, 1♂; Lushan, 27.VI–5.VII.1975, coll. Liu Youqiao, 1♂1♀; Dayu, 550 m, 15.VIII.1985, coll. Wang Ziqing, 1♂. **Hunan** (IZCAS):Yizhang, 27.VII.1981, 1♂; Yizhang, Mangshan, Senlin Gongyuan, 512–770 m, 13–15.VII.2008, coll. Chen Fuqiang, 1♂; Yongshun, Shanmuhe Linchang, 500–600 m, 3–5.VIII.1998, coll. Chen Yixin et al., 8♂4♀; Guidong, Sidu, 714 m, 9–12.VII.2008, coll. Chen Fuqiang, 1♂; Yanling, Taoyuandong, 631 m, 4–8.VII.2008, coll. Chen Fuqiang, 2♂; Anhua, 22.V.1981, 1♂; Guzhang, Gaowangjie, 850 m, 29.VII.1988, coll. Chen Yixin, 2♂; Fenghuang, 16.IX.1988, coll. Song Shimei, 1♂. **Fujian** (IZCAS): Mt. Wuyi, Sangang, 10.V–5.VI.1983, coll. Zhang Baolin et al., 13♂3♀; Mt. Wuyi, 500–700 m, 25–29.VII.2006, coll. Yang Chao et al., 4♂; Mt. Wuyi, Sangang, 704 m, 11–14.VIII.2009, coll. Jiang Nan and Xue Dayong, 8♂; Ninghua, 9.VI.1980, 1♂; Sanming, 1.IX.1981, coll. Xiao Hu, 1♂; Jiangle, Longqishan, 200 m, 10.VIII.1991, coll. Song Shimei, 1♂; Shaxian, 3.IX.1979, coll. Lin Naiquan, 1♂. **Guangdong** (IZCAS): Dinghushan, 16–19.VII.1979, coll. Li Mingjia, 1♂; Luyuan, Nanling, 1020 m, 16–20.VII.2008, coll. Chen Fuqiang, 3♂; Shixing, Chebaling, 365–401 m, 22–26.VII.2008, coll. Chen Fuqiang, 15♂1♀. **Hainan** (IZCAS): Qiongzhong, Limuling, 620 m, 14–15.V.2007, coll. Han Hongxiang and Lang Songyun, 4♂; Baisha, Yinggeling, 434 m, 5.IV.2008, coll. Lang Songyun, 1♂; Baisha, Hongkan, Shuiku, 553 m, 3–5.V.2009, coll. Yan Keji, 2♂; Wuzhishan, 29.V.1997, coll. Mai Guoqing, 1♂; Wuzhishan, Shuiman, 730–900 m, 3.IV–9.V.2007, coll. Han Hongxiang and Lang Songyun, 3♂; Ledong, Jianfengling, Tianchi, 982 m, 23.XI.2008, coll. Li Jing, 1♂; Lingshui, Diaoluoshan, 920 m, 29–30.III.2008, coll. Lang Songyun, 4♂. **Guangxi** (IZCAS): Longsheng, Hongtan, 900 m, 14.VI.1963, coll. Wang Chunguang, 1♂; Mao’ershan, Jiuniutang, 1100–1150 m, 6–10.VII.1985, coll. Fang Chenglai, 2♂; Jinxiu, 400–1100 m, 10–18.V.1999, coll. Han Hongxiang et al., 69♂4♀. **Sichuan** (IZCAS):Guanxian, Dujiangyan, 700–1000 m, 4.VI.1979, coll. Shang Jinwen, 1♂; Mt. Emei, Qingyinge, 800–1000 m, 23.IV–16.VII.1957, coll. Zhu Fuxing, 38♂; Jiangjin, Simianshan, 22–26.VI.1981, coll. Zhang Shuli, 2♂; Pengshui, Taiyuan, 750 m, 9.VII.1989, coll. Yang Longlong, 2♂; Wanxian, Wangerbao, 1200 m, 28.V.1994, coll. Li Wenzhu, 2♂. **Guizhou** (IZCAS): Jiangkou, Fanjingshan, 500 m, 11.VII.1988, coll. Li Wei, 12♂; Leishan, Leigongshan, 1200 m, 3.VII.1988, coll. Yuan Decheng, 1♀; Shiyun, Jinxing, 700 m, 24.VII.1988, coll. Wang Shuyong, 1♂1♀. **Yunnan** (IZCAS):Baoshan, Baihualing, 1520 m, 11–13.VIII.2007, coll. Wu Chunguang and Xue Dayong, 2♂; Baoshan, Bawan, 1040 m, 8–10.VIII.2007, coll. Xue Dayong, 1♂; Xishuangbanna, Bubang, 700 m, 14.IX.1993, coll. Yang Longlong, 1♂. **Tibet** (IZCAS): Zayü, Xia Zayü, 1534 m, 26.VIII.2005, coll. Wang Xuejian, 1♂1♀; Mêdog, 871–2095 m, 10–23.VIII.2006, coll. Lang Songyun, 8♂3♀. A large series from many parts of China in coll. ZFMK.

#### Distribution.

China (Liaoning, Beijing, Hebei, Shanxi, Shandong, Henan, Shaanxi, Ningxia, Gansu, Anhui, Zhejiang, Hubei, Jiangxi, Hunan, Fujian, Guangdong, Hainan, Guangxi, Sichuan, Guizhou, Yunnan, Tibet), India, Nepal, Sikkim, Vietnam, Thailand.

#### Remarks.

For *Biston panterinaria* six subspecies have been described: *Biston panterinaria panterinaria* (north China), *Biston panterinaria abraxata *(Jiangxi, China), *Biston panterinaria lienpingensis *(Guangdong, China), *Biston panterinaria szechuanensis *(Sichuan, China), *Biston panterinaria sychnospilas *(Prout, 1930) (Japan) and *Biston panterinaria exanthemata *(Moore, 1888) (India, Nepal, Vietnam and Thailand). Sato (1996) believed that there are geographical variations among *Biston panterinaria exanthemata *in India, Nepal, Sikkim, Vietnam and Thailand. These variations can be found in the appearance and in the male genitalia. He also believed that the four Chinese subspecies exhibit intermediate conditions in colour and patches on wings between *Biston panterinaria szechuanensis* and *Biston panterinaria exanthemata*, and the systematic position of the Chinese populations of *Biston panterinaria* needs further study (Sato 1996). According to the descriptions of Wehrli (1939), ssp. *abraxata* has distinct yellow postmedial lines accompanied by distinct dark brown patches as above, ssp. *lienpingensis* has the weak postmedial line accompanied with dark brown patches, ssp. *szechuanensis* has well developed grey markings. However, we consider these differences to be actually intraspecific individual variations. The yellow postmedial lines, the brown patches and the grey markings vary and are differently developed not only among the different populations, but often occur in the same region. For instance, the yellow postmedial line varies from distinct and complete (ssp. *abraxata*) to incomplete (ssp. *lienpingensis*), and the variation occurred sympatrically in Jiangxi (Figs 60, 61). The material with well developted grey markings on the wings (ssp. *szechuanensis*) and the material without well developted grey markings on the wings (ssp. *panterinaria*) occurred sympatrically in Beijing (Figs 64, 65). What is more, the similar variation occurs in different localities. For example, the form with distinct and almost continuous yellow postmedial line (ssp. *abraxata*) occurs in Beijing (Fig. 65), besides Jiangxi (Fig. 60); the form similar to the subspecies *lienpingensis* occurs in Guangdong (Fig. 62), as well as in Zhejiang (Fig. 58) and Sichuan (Fig. 63); the form with well developed grey markings (ssp. *szechuanensis*) occurs in Beijing (Fig. 64), besides Sichuan (Fig. 59). In the male genitalia, the sinuous ventral margins of the valva vary from strongly to weakly among the material from different localities (Figs 90, 91, 92, 93), or even from the same region, such as Chebaling, Shixing of Guangdong (Figs 94, 95). Thus, we regard the variations as intraspecific individual variations, and treat *Biston panterinaria lienpingensis*, *Biston panterinaria szechuanensis* and *Biston panterinaria abraxata *as synonyms of *Biston panterinaria panterinaria*. Besides, we find that the material collected from Yunnan and Tibet is identical with *Biston panterinaria exanthemata* which was redescribed and illustrated by Sato (1996). Hence, we classify the material from Yunnan and Tibet as *Biston panterinaria exanthemata* (Figs 66–69, 96, 116, 130)(and new to the fauna of China).

**Figures 82–88.**
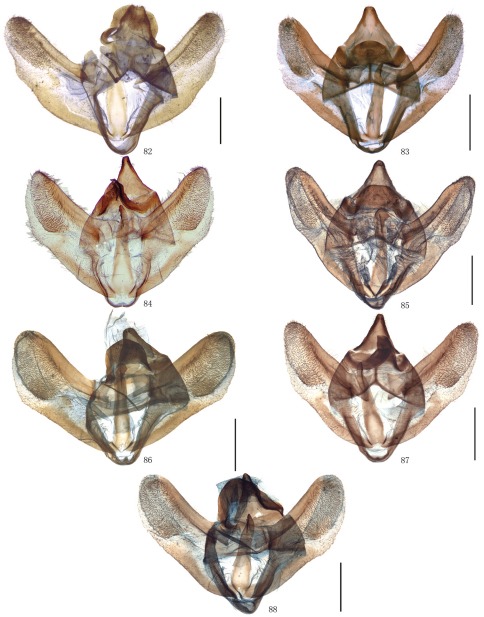
Male genitalia of *Biston*. **82** *Biston brevipennata* **83** *Biston quercii* **84–88**. *Biston falcata* **84** *Biston falcata falcata* (holotype of *Amphidasis erilda*) **85** *Biston falcata* *falcata* (Nyalam, Tibet) **86** *Biston falcata falcata *(Mainling, Tibet) **87** *Biston falcata satura *(Zhouqu, Gansu) **88** *Biston falcata satura* (Shalin, Gansu). Scale bar = 1 mm.

**Figures 89–94.**
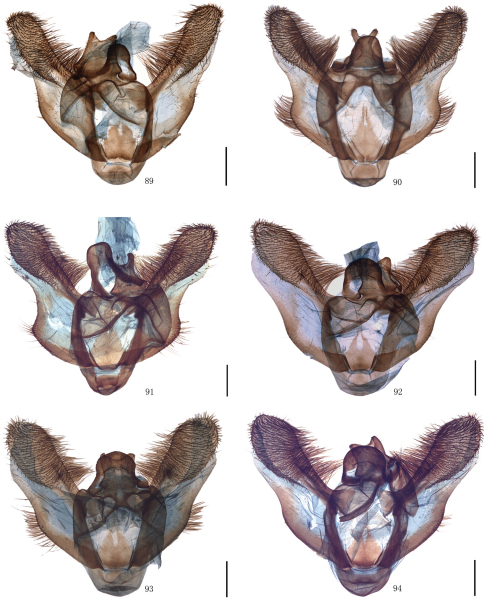
Male genitalia of *Biston*. **89** *Biston thibetaria*; **90**–**94**. *Biston panterinaria panterinaria*. **90** From Sanpu, Beijing **91** From Dayu, Jiangxi **92** From Wuyanling, Taishun, Zhejiang **93** From Taiyuan, Pengshui, Sichuan **94 **From Chebaling, Shixing, Guangdong. Scale bar = 1 mm.

**Figures 95–104.**
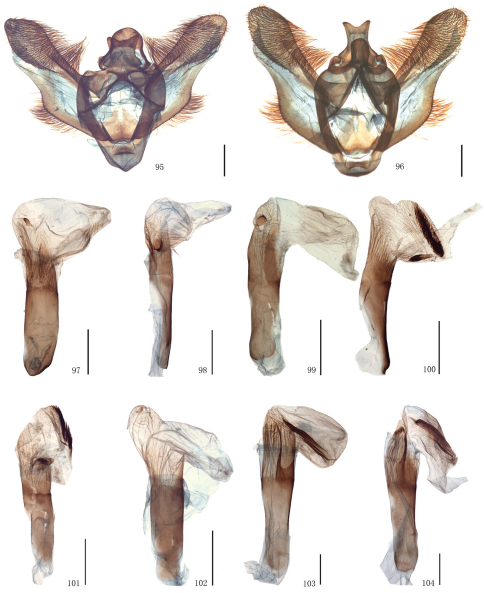
Male genitalia of *Biston*. **95** *Biston panterinaria panterinaria*. From Chebaling, Shixing, Guangdong **96** *Biston panterinaria exanthemata*. Scale bar = 1 mm. Aedeagus of *Biston*. **97** *Biston melacron* **98** *Biston marginata* **99** *Biston thoracicaria* **100** *Biston betularia parva* **101** *Biston betularia nepalensis* **102** *Biston robustum* **103** *Biston mediolata* sp. n. **104** *Biston contectaria*. Scale bar = 1 mm.

**Figures 105–116.**
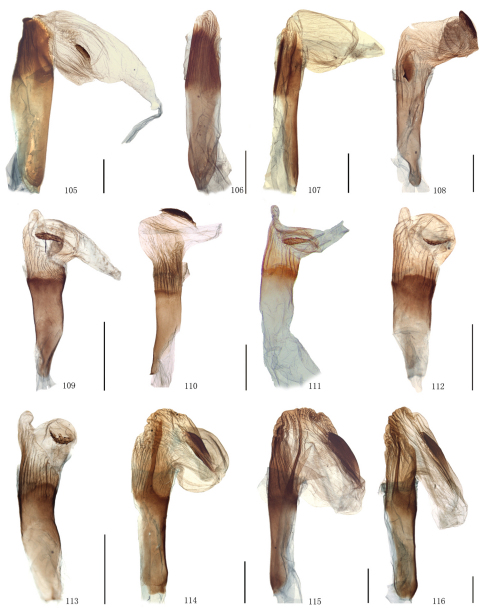
Aedeagus of *Biston*. **105** *Biston bengaliaria* **106** *Biston pustulata* **107** *Biston suppressaria* **108** *Biston regalis* **109** *Biston brevipennata* **110** *Biston quercii* **111** *Biston falcata falcata* (holotype of *Amphidasis erilda*) **112** *Biston falcata falcata* **113** *Biston falcata satura* **114** *Biston thibetaria* **115** *Biston panterinaria panterinaria* **116** *Biston panterinaria exanthemata*.Scale bar = 1 mm.

**Figures 117–120.**
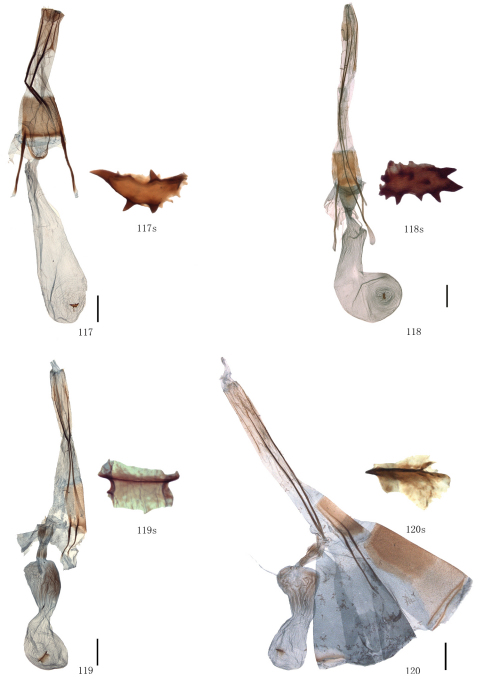
Female genitalia of *Biston* and enlarged view of signum. **117** *Biston marginata* **118** *Biston thoracicaria* **119** *Biston betularia parva* **120** *Biston betularia nepalensis*. Scale bar for female genitalia = 1 mm. (s = signum)

**Figures 121–124.**
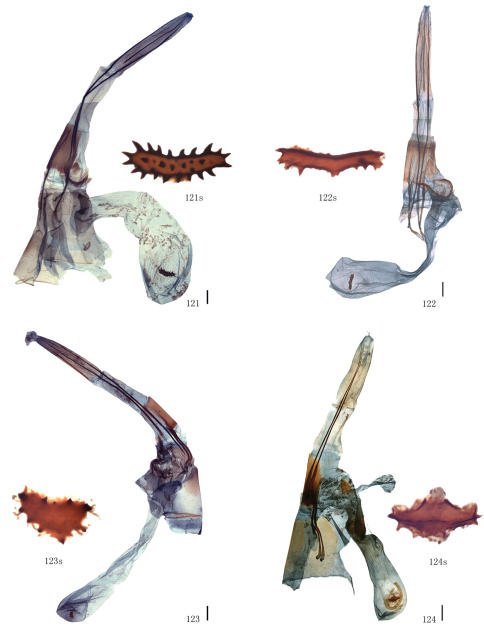
Female genitalia of *Biston* and enlarged view of signum. **121** *Biston mediolata* sp. n. **122** *Biston bengaliaria* **123** *Biston suppressaria* **124** *Biston regalis*. Scale bar for female genitalia = 1 mm. (s = signum)

**Figures 125–128.**
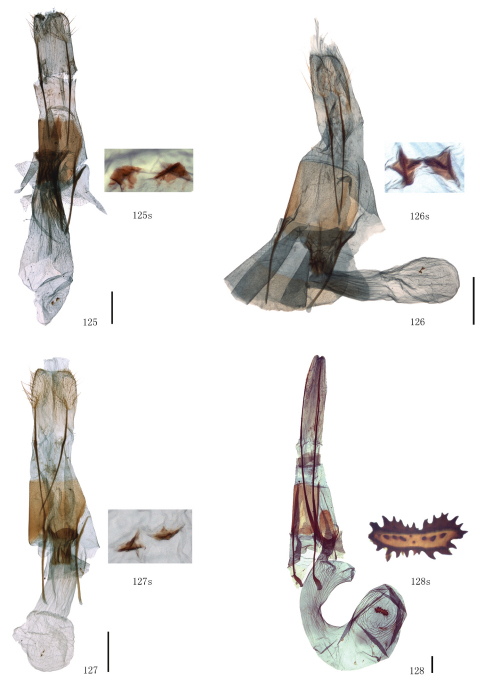
Female genitalia of *Biston* and enlarged view of signum. **125** *Biston falcata* *falcata* (Diqing, Yunnan) **126** *Biston falcata* *falcata* (Gyirong, Tibet) **127** *Biston falcata* *satura* (Zhouqu, Gansu) **128** *Biston thibetaria*. Scale bar for female genitalia = 1 mm. (s = signum)

**Figures 129–130.**
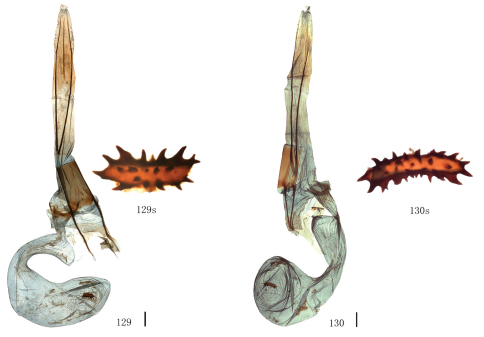
Female genitalia of *Biston* and enlarged view of signum. **129** *Biston panterinaria panterinaria* **130** *Biston panterinaria exanthemata*. Scale bar for female genitalia = 1 mm. (s = signum)

## Supplementary Material

XML Treatment for Biston

XML Treatment for Biston melacron

XML Treatment for Biston marginata

XML Treatment for Biston thoracicaria

XML Treatment for Biston betularia

XML Treatment for Biston robustum

XML Treatment for Biston mediolata

XML Treatment for Biston contectaria

XML Treatment for Biston bengaliaria

XML Treatment for Biston pustulata

XML Treatment for Biston suppressaria

XML Treatment for Biston regalis

XML Treatment for Biston brevipennata

XML Treatment for Biston quercii

XML Treatment for Biston falcata

XML Treatment for Biston perclara

XML Treatment for Biston thibetaria

XML Treatment for Biston panterinaria
